# From Oncolysis to Adaptive Immunity: Yellow Fever Virus 17D and Ruxolitinib Activate Antitumor Immune Responses in Pancreatic Cancer Models

**DOI:** 10.3390/biomedicines14081763

**Published:** 2026-08-05

**Authors:** Kirill N. Trachuk, Yulia K. Biryukova, Vitalii A. Kapranov, Alina S. Nazarenko, Ekaterina A. Orlova, Grigory L. Kozhemyakin, Grigory A. Demyashkin, Ilya V. Gordeychuk, Aydar A. Ishmukhametov, Nadezhda M. Kolyasnikova

**Affiliations:** 1 Laboratory of Tick-Borne Encephalitis and Other Viral Encephalitides, Chumakov Federal Scientific Center for Research and Development of Immune-and-Biological Products of Russian Academy of Sciences (Institute of Poliomyelitis), 108819 Moscow, Russiakolyasnikova_nm@chumakovs.su (N.M.K.); 2 Scientific Research Institute for Systems Biology and Medicine, 117246 Moscow, Russia; 3 Institute for Translational Medicine and Biotechnology, Sechenov University, 119048 Moscow, Russia; demyashkin_g_a@staff.sechenov.ru

**Keywords:** virotherapy, cancer immunotherapy, PDAC, 17D, yellow fever vaccine, ruxolitinib

## Abstract

**Background**: Pancreatic ductal adenocarcinoma (PDAC) remains one of the most lethal cancers, with a five-year survival rate of less than 12%. Oncolytic viruses are considered a promising immunotherapeutic approach, but their effectiveness is often limited by the innate interferon response, which is the main antiviral barrier in resistant tumors. A combination of an oncolytic virus with JAK/STAT pathway inhibitors has been proposed to overcome this resistance. **Methods**: In this study, we investigated the oncolytic and immunotherapeutic potential of the attenuated yellow fever vaccine strain YFV 17D in combination with the JAK1/JAK2 inhibitor ruxolitinib in a panel of six PDAC cell lines with diverse genetic profiles and JAK/STAT signaling activities and in a syngeneic immunocompetent PAN02 mouse model. **Results**: In vitro, we identified three distinct response patterns: synergistic enhancement of viral replication and cytopathic effect, lack of synergism due to absent interferon signaling, and increased viral replication without cytopathic effect. Despite different cell mutation profiles, the cell response patterns were determined by the basal JAK/STAT activity of each cell line rather than by specific *KRAS* or *TP53* mutation. In vivo, the combination therapy significantly extended median survival compared to YFV 17D monotherapy and the control group and induced tumor regression in 62.5% of animals. Since no difference in intratumoral viral RNA was detected between the combination and monotherapy groups, we suggest that the antitumor effect in vivo was mediated not by enhanced viral replication, but by activation of adaptive immunity. This is indirectly suggested by increased intratumoral IL-12, a fourfold increase in CD8^+^ T cell infiltration, a reduction in CD4^+^ cells, and the generation of virus-neutralizing antibodies. No systemic cytokine surge, neurovirulence, or viral dissemination was observed, suggesting the safety of the proposed regimen. **Conclusions**: These findings clarify the immune mechanisms determining the efficacy of YFV 17D combined with ruxolitinib and establish the basis for personalized patient stratification by tumor interferon signaling status.

## 1. Introduction

Despite advances in chemotherapy, radiotherapy, and targeted therapy, pancreatic ductal adenocarcinoma (PDAC) remains one of the most lethal cancers, with a five-year survival rate of less than 12%. The reasons are its rapid progression, early metastasis, and multiple mechanisms conferring the ability to rapidly develop resistance to conventional treatment strategies. PDAC is characterized by a high mutation rate, with more than 95% of the tumors containing mutations upregulating transcription of the *KRAS* gene. This is considered one of the key events leading to carcinogenesis [1]. Genetic profiles of approximately 70% of PDACs show them having mutations in the *TP53* gene encoding the p53 tumor suppressor protein and mutations inactivating transcription of the *CDKN2A* (p16) and *SMAD4* genes [2]. In conjunction, these genetic variations form specific signatures determining main characteristics of the aforementioned tumor cells, including their basal JAK/STAT signaling pathway activity, interferon sensitivity, susceptibility to apoptosis, and viral permissivity [3]. Thus, the interaction between high transcriptional activity of the *KRAS* gene and the mutant p53 protein leads to hyperexpression of transcriptional factors that promote metastasis and invasiveness of the tumor [4].

**Figure 1:**
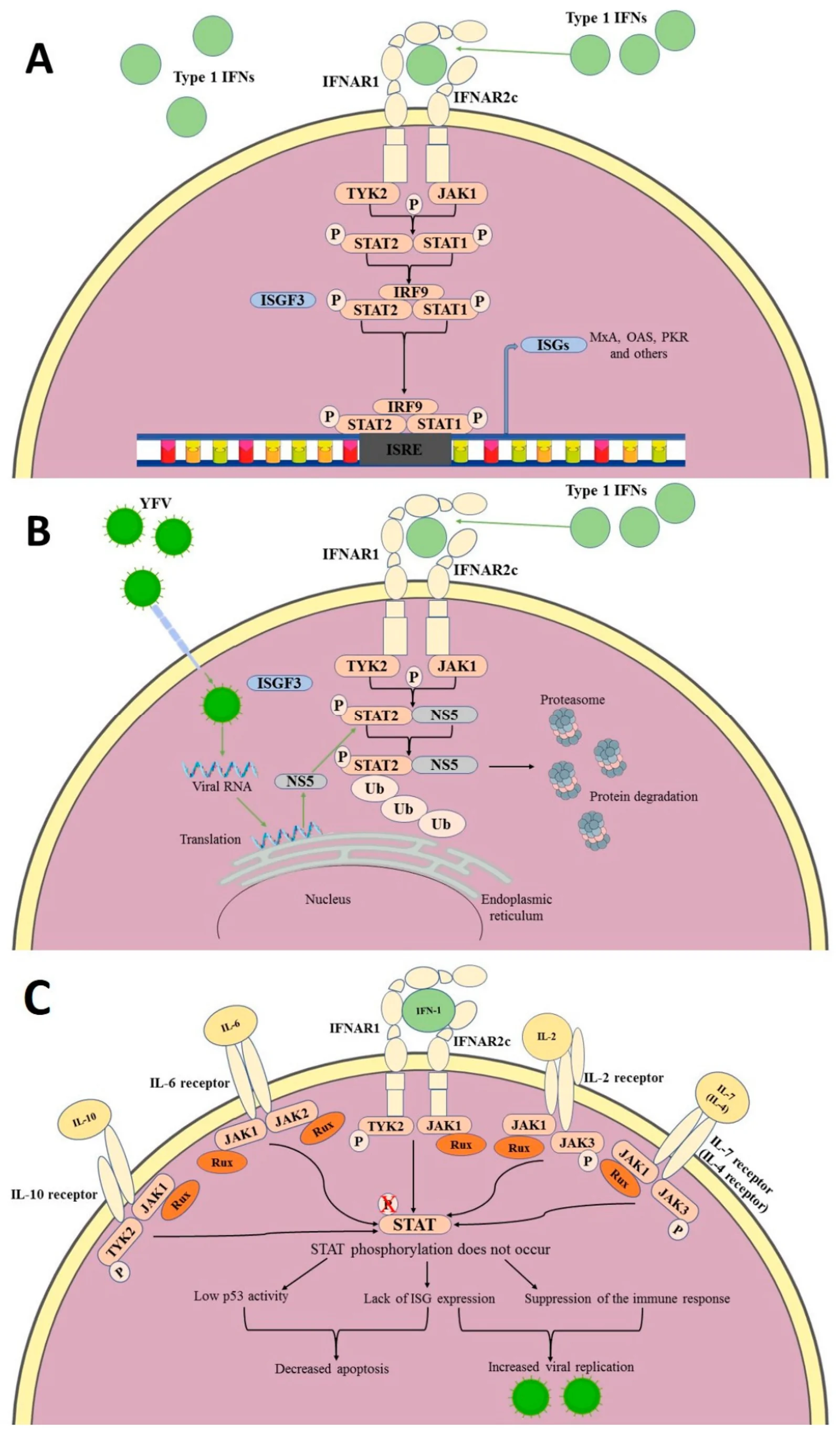
(**A**) The type I IFN signaling pathway. Type I IFN interacts with IFNAR1 and IFNAR2c receptors, activating the JAK/STAT signaling pathway. TYK2 and JAK1 tyrosine kinases phosphorylate STAT1 and STAT2 proteins, which are then joined by the transcription factor IRF9, forming the ISGF3 signaling complex. The ISGF3 then binds to the ISRE promoter region, triggering ISG expression. (**B**) Inhibition of the type I IFN signaling pathway by the NS5 protein. IFN type I interacts with the IFNAR1 and IFNAR2c receptors, and TYK2 and JAK1 phosphorylate STAT2. Then STAT2, which would normally be bound by STAT1, is instead bound by the viral protein NS5. This leads to polyubiquitination of the STAT2-NS5 complex and its proteasomal degradation. As a result, ISG expression does not occur, allowing the virus to replicate. (**C**) Inhibition of the JAK/STAT signaling pathway by ruxolitinib. Ruxolitinib, competitive to ATP, binds to JAK1 and JAK2, preventing phosphorylation of STAT proteins. Inhibition of JAK kinases leads to the inhibition of many STAT-mediated pathways, ultimately leading to immunosuppression, decreased ISG expression, decreased p53 activity, and other associated events. It should be noted that the figure shows only a subset of the receptors that activate the JAK/STAT pathway.

Currently, oncolytic virotherapy has been considered a promising approach capable of increasing the effectiveness of conventional cancer therapy due to selective lysis of tumor cells and induction of antitumor immunity [5,6]. The strain 17D of yellow fever virus (YFV 17D), known for its safety as a vaccinal strain and its ability to elicit a strong immune response, has the potential for use as an oncolytic agent [7]. YFV 17D has an extremely low risk of reversion to a wild-type virulence profile due to 68 nucleotide substitutions and 32 amino acid changes distributed throughout its genome relative to the parent strain Asibi—a genomic basis for attenuation that has precluded any documented cases of reversion in over 80 years of vaccine use. The antiviral state of the tumor, including interferon-mediated mechanisms, is considered the key challenge, overcoming which is crucial for increasing the effectiveness of oncolytics. One of the most important mechanisms of flavivirus (including YFV) evasion of the immune system is dependent on the presence of the viral nonstructural protein 5 (NS5) [8]. It specifically binds to the human STAT2 protein (hSTAT2), promoting its polyubiquitination and degradation through the proteasome, which blocks the type I interferon signaling pathway and interferon-stimulated gene (ISG) production (Figure 1B). This antagonism is species-specific: the NS5 protein effectively degrades hSTAT2 but lacks the ability to bind to murine STAT2 (mSTAT2) [9]. Hence, the NS5-STAT2 interaction causes a fundamental difference in viral permissivity between mouse and human cancer cell lines. Due to this, PAN02 was included as a species-specific control for the NS5-STAT2 antagonism.

The JAK/STAT signaling pathway is an important component of the innate immune response and the main obstacle limiting the effectiveness of oncolytics in resistant tumors [10]. JAK/STAT-dependent expression of ISGs, especially *MxA*, *OAS*, and other genes encoding antiviral proteins, provides protection against viral infection and virus replication. When activated by interferon type I (IFN), JAK kinases phosphorylate the STAT1 and STAT2 proteins, promoting their heterodimerization with the IRF9 protein and formation of the ISGF3 complex. This complex subsequently translocates into the nucleus and activates ISG expression (Figure 1A). Therefore, activation of this pathway leads to the development of tumor resistance to oncolytic viruses. Previous studies have shown that selective JAK inhibitors, especially ruxolitinib (a JAK1/JAK2 inhibitor), significantly enhance viral replication in resistant PDAC cell lines by suppressing IFN type I production and the subsequent ISG expression (Figure 1C) [11,12].

In this study, we sought to overcome one of the major challenges of oncolytic virotherapy, the tumor interferon barrier, by using controlled administration of ruxolitinib to enhance the oncolytic effect of YFV 17D. This approach was first tested in vitro using a panel of six well-described PDAC cell lines with different genetic profiles (key characteristics are presented in Table 1). We identified three distinct patterns of response to the combination used: synergism, lack of synergism, and increased the virus’ replicative activity without a cytopathic effect. Proceeding further, we investigated the immunotherapeutic potential of the combination of YFV 17D with ruxolitinib in vivo using a syngeneic immunocompetent PAN02 model in C57BL/6 mice using a short-term ruxolitinib administration regimen. We analyzed changes in tumor growth dynamics, animal survival, and antiviral and antitumor immune response in mice, as well as the safety of this combination therapy.

## 2. Materials and Methods

### 2.1. Cell Lines

Vero monkey kidney epithelial cells (ATCC CCL-81) were obtained from the American Cell Culture Collection (ATCC); human pancreatic carcinoma cell lines MIA PaCa-2, PANC-1, AsPC-1, Capan-2, BxPC-3, and murine pancreas adenocarcinoma PAN02 were obtained from the collection of the Laboratory of Tick-Borne Encephalitis and Other Viral Encephalitides (Chumakov FSC R&D IBP RAS (Institute of Poliomyelitis)). All cell lines used in the work were negative for mycoplasma (mycoplasma detection kit Myco Real-Time, Evrogen (Moscow, Russia), cat. No. MR004). All human cancer cell lines used in this study were authenticated by short tandem repeat (STR) profiling and were routinely tested to confirm their identity and to exclude cross-contamination.

Cells were cultured in Dulbecco’s Modified Eagle Medium (DMEM; Chumakov FSC R&D IBP RAS (Institute of Poliomyelitis)) supplemented with 5–10% fetal bovine serum (FBS; Gibco (Grand Island, NY, USA) cat. No. 2412072), 2 mM L-glutamine (Paneco (Singapore), cat. No. F032), and 50 µg/mL penicillin–streptomycin at 37 °C in a humidified atmosphere containing 5% CO_2_. As a supporting medium after virus inoculation, we used the Improved Minimum Essential Medium (IMEM; Chumakov FSC R&D IBP RAS (Institute of Poliomyelitis)) with the identical additives but 2% FBS.

### 2.2. Cultivation and Quantification of YFV 17D

We used the attenuated vaccine strain YFV 17D from the collection of Chumakov FSC R&D IBP RAS (Institute of Poliomyelitis). The virus was propagated in Vero cells and further clarified by centrifugation. The YFV 17D stock intended for in vivo use was additionally purified and resuspended in PBS. The virus titer was assessed by the plaque assay. Briefly, serial tenfold dilutions of 17D YFV were inoculated into a monolayer of Vero cells in 12-well plates and incubated for 60 min in an atmosphere of 5% CO_2_ at 37 °C. Following this, 1 mL of coating was added. The coating composition was medium 199 with Earle and Hanks salts (Chumakov FSC R&D IBP RAS (Institute of Poliomyelitis)) in a 2:1 ratio, 1.25% methylcellulose (Merc), 0.1% gentamicin (Paneco cat. No. A011), and 2% FBS. Seven days post-infection, the coating was removed, cells were washed with PBS, fixed with 95% ethanol, and stained with 0.1% crystal violet dye (Sigma-Aldrich, St. Louis, MO, USA). The number of plaques for each dilution was then counted to estimate plaque-forming units per mL (PFU/mL).

### 2.3. xCELLigence Real-Time Cell Proliferation Measurement

Cell proliferation was monitored using Agilent’s Real-Time Cell Analysis (RTCA) system. Tumor cells were cultured in T25 flasks (25 cm^2^) in DMEM supplemented with 5–10% FBS, 2 mM L-glutamine, and 50 µg/mL penicillin–streptomycin at 37 °C in a humidified atmosphere with 5% CO_2_ until 70–80% confluence. The culture medium was then aspirated from both flasks. One flask received 2 mL of fresh DMEM containing 1 µM ruxolitinib (BLD Pharm (Shanghai, China), cat. no. BD165439, CAS 941678-49-5), while the other received 2 mL of drug-free DMEM. Cells were incubated for 3 h at 37 °C and 5% CO_2_.

Following pre-treatment, cells were detached using 0.25% trypsin-EDTA in Hanks’ balanced salts (PanEco, Cat. No. P041), resuspended in DMEM with or without 1 µM ruxolitinib, and aliquoted into four tubes. Two tubes were inoculated with YFV 17D at a multiplicity of infection (MOI) of 1.0; control tubes received an equivalent volume of DMEM. Cells were then seeded onto E-Plate 16 (Agilent xCELLigence RTCA system, Santa Clara, CA, USA) at a density of 1 × 10^6^ cells/mL, adding 100 µL of the cell suspension per well according to the experimental layout. The plates were incubated at 37 °C in a humidified atmosphere of 5% CO_2_, and real-time cell index (proliferation/impedance) was recorded every 2 h for 7 days.

### 2.4. RNA Extraction and Quantitative RT-PCR

The presence of viral RNA in the culture medium, tumor homogenates, brains, and spleens was assessed using a quantitative PCR method. A 0.1 mL sample of culture medium was collected, and cell debris was removed by centrifugation at 3000 rpm for 5 min. Samples of tumors, brains, and spleens were homogenized, resuspended in PBS, treated with lysis solution, and then centrifuged at 7000 rpm for 5 min. Viral RNA was isolated from the supernatant using the Ribo-prep reagent kit according to the manufacturer’s instructions. PCR was performed using the AmpliSens Yellow Fever Virus-FL reagent kit and a set of calibrators. All reagent kits were produced by the Central Research Institute of Epidemiology of Rospotrebnadzor (Moscow, Russia). Quantitative real-time PCR was conducted on a CFX96 (Bio-Rad) system using the FAM and HEX detection channels, and the samples were interpreted as positive or negative for yellow fever virus RNA according to the kit guidelines.

To determine the titer of infectious 17D virus in the collected tumors, tumor exudates that had previously been analyzed by PCR were used to infect the Vero cells, followed by incubation for 5 days, after which the culture fluid was collected and analyzed by PCR for the presence of 17D RNA.

### 2.5. Virus Titer Evaluation

Tumor cells were cultured in 12-well plates in DMEM supplemented with 5–10% FBS, 2 mM L-glutamine, and 50 µg/mL penicillin–streptomycin at 37 °C and 5% CO_2_ until they reached 70–80% confluence. The culture medium was then aspirated, and 400 µL of DMEM containing ruxolitinib at a final concentration of 1 or 5 µM was added to each well according to the experiment scheme. Control wells received 400 µL of DMEM alone. Cells were incubated for 3 h at 37 °C and 5% CO_2_.

Following this, 400 µL of a suspension containing YFV 17D (the inoculum) at a titer of 2 × 10^7^ PFU/mL was added to each well (excluding control wells, to which an equivalent volume of DMEM was added). The plates were incubated with continuous gentle agitation for 1 h at 37 °C and 5% CO_2_. The inoculum was then aspirated, and 2 mL of fresh DMEM containing ruxolitinib at 1 or 5 µM was added to each well (excluding control wells, to which an equivalent volume of DMEM was added). The cells were then incubated for 7 days under standard culture conditions. Cytopathic effect (CPE) was evaluated daily by light microscopy, and culture supernatant from wells with the virus added was collected for virus titer evaluation by plaque assay and qualitative real-time PCR, which were performed according to protocols described previously.

### 2.6. Annexin V–FITC FLUORESCENCE Microscopy

Apoptosis in Capan-2 and AsPC-1 cells was assessed using the Annexin V–FITC apoptosis detection kit (BD FITC APO Detection Kit II (BD Biosciences, Milpitas, CA, USA), cat. no. 556570) according to the manufacturer’s instructions, with minor modifications. Cells were seeded in 12-well plates and cultured as described in the “Virus titer evaluation” section. On days 5 and 7 post-treatment, the culture medium was aspirated from two wells per condition, and the cells were washed twice with PBS. A working staining solution was prepared by diluting FITC-conjugated Annexin V 1:10 in Annexin V binding buffer (100 µL FITC Annexin V mixed with 900 µL binding buffer for two wells). Then, 500 µL of the staining solution was added to each well, and the plates were incubated for 15 min at room temperature in the dark. After incubation, the staining solution was aspirated, the cells were washed once with Annexin V binding buffer, and 500 µL of fresh binding buffer was added to each well. Samples were immediately examined using a fluorescence microscope at ×100 magnification with a FITC filter set (excitation ~488 nm, emission ~520 nm). Cells displaying membrane-associated FITC fluorescence were considered Annexin V-positive, indicative of phosphatidylserine externalization and apoptosis.

### 2.7. In Vivo Experiments

#### 2.7.1. Animals and Ethical Statement

Female C57BL/6 mice (6–8 weeks of age, body weight 18–22 g) were obtained from the SPF vivarium of the Institute of Cytology and Genetics SB RAS (Novosibirsk, Russia) and housed under specific pathogen–free conditions (12 h light/dark cycle, controlled temperature and humidity) with ad libitum access to food and water. All procedures were performed in accordance with national guidelines for the care and use of laboratory animals and were approved by the Ethics Committee of Chumakov FSC R&D IBP RAS (Institute of Poliomyelitis) (protocol No. 28082025).

#### 2.7.2. Tumor Cell Line and Its Implantation

The murine pancreatic ductal adenocarcinoma cell line PAN02 (C57BL/6 background) was cultured in DMEM supplemented with 5–10% FBS, 2 mM L-glutamine, and 50 μg/mL penicillin–streptomycin at 37 °C in a humidified 5% CO_2_ atmosphere as described above for in vitro experiments. For tumor implantation, PAN02 cells in the logarithmic growth phase were harvested by trypsinization, washed twice in sterile PBS, counted, and resuspended at 2 × 10^7^ cells/mL. Mice were anesthetized with isoflurane, and 2 × 10^6^ PAN02 cells in a total volume of 100 μL of PBS were injected subcutaneously into the lateral aspect of the left thigh.

Tumor growth was monitored three times per week using a digital caliper, and tumor volume was calculated using the formula V = (L × W^2^)/2, where «L» is the longest diameter and «W» is the shortest diameter. Treatment was initiated when tumors reached a diameter of approximately 5 mm (~80–120 mm^3^). The mice were monitored for signs of distress, and humane endpoints were predefined as any of the following: tumor volume ≥ 500 mm^3^, impaired mobility, or >15% loss of baseline body weight. The mice reaching a humane endpoint were euthanized by isoflurane inhalation followed by cervical dislocation. Tumor growth curves were plotted for individual animals for each group. Overall survival was analyzed using the Kaplan–Meier method, and differences between groups were assessed by the log-rank (Mantel–Cox) test (GraphPad Prism v8.2). Median survival times were calculated along with 95% confidence intervals.

#### 2.7.3. Randomization and Treatment Groups

When tumors reached the predefined size, mice were randomly assigned to four experimental groups (n = 8 per group) to evaluate the antitumor efficacy of YFV 17D and its combination with ruxolitinib:

1. Vehicle control (mock): intratumoral (i.t.) injection of 100 μL sterile PBS.
2. Ruxolitinib control: i.p. ruxolitinib with i.t. PBS.
3. YFV 17D monotherapy: single i.t. injection of 1 × 10^5^ PFU of YFV 17D in 100 μL.
4. Combination therapy (YFV 17D with ruxolitinib): i.p. ruxolitinib was administered 24 h and 3 h before i.t. administration of YFV 17D, and again 24 and 48 h after virus injection, plus a single i.t. dose of 1 × 10^5^ PFU YFV 17D in 100 μL was administered on day 0.

Ruxolitinib (BLD Pharm, cat. no. BD165439, CAS 941678-49-5) was prepared at a final concentration of 10 mg/mL in a vehicle containing 10% DMSO, 40% PEG300, 5% Tween-80, and PBS. The prepared solution was administered i.p. at 100 μL per dose.

#### 2.7.4. Collection of Tumor and Organ Samples

For ELISA, IHC, and PCR-RT analysis, subgroups of mice were sacrificed according to the experiment design. Tumors were excised, weighed, and used for RNA extraction, cytokine measurement, and planned IHC. Tumor samples for RNA and cytokines were snap-frozen in liquid nitrogen and stored at −80 °C until processing, whereas samples for histological analysis were fixed in 10% neutral-buffered formalin and embedded in paraffin. To assess systemic safety and virus dissemination, brains and spleens were collected from all mice treated with YFV 17D (both monotherapy and combination groups) at the time of sacrifice. Tumor tissues were homogenized in PBS, and the aliquots were used for quantitative RT-PCR and for passaging on Vero cell monolayers.

#### 2.7.5. Cytokine Analysis in Tumor Homogenates

To characterize the local immune response, concentrations of IFN-α, TNF-α, and IL-12 were measured in tumor homogenates collected on days 3, 10, and 25+ after therapy initiation (n = 4 per group per time point). Tumors were excised on days 3, 10, and 25+ after therapy, briefly rinsed in ice-cold PBS, weighed, and snap-frozen at −80 °C. For homogenization, frozen tumors were ground in pre-chilled mortars with liquid nitrogen and resuspended on ice in NP-40 lysis buffer supplemented with protease inhibitors (1:1000). Tumor lysates were incubated for 30 min at 4 °C with gentle mixing applied and centrifuged at 10,000 g for 10 min; supernatants were collected and either used immediately or stored at −80 °C. Cytokine levels were quantified using commercial ELISA kits specific for murine IFN-α, TNF-α, and IL-12 (Cloud-Clone Corp (Katy, TX, USA), cat. no. IEA033Mu, SEA133Mu, SEA058Mu) according to the manufacturers’ protocols and normalized to total protein content. Cytokine concentrations are reported as pg per mg of total protein.

Blood was collected from anesthetized mice into tubes with a clot activator, allowed to clot for 60 min at room temperature, centrifuged at 8000× *g* for 5 min, and blood serum was aliquoted and stored at −80 °C until use. Importantly, the measurement of the same cytokines in serum did not yield values above the detection limits of the assays in any group, indicating the absence of a systemic cytokine surge.

#### 2.7.6. Neutralizing Antibody Assay

To analyze titers of 17D-neutralizing antibodies in mice, we used a plaque assay method. Immediately before sacrificing mice, we collected sera from their blood. The sera were then diluted in serial three-fold dilutions and mixed with 17D (3 × 10^3^ PFU/mL), followed by incubation for 1 h at 37 °C. YFV 17D (1.5 × 10^3^ PFU/mL) was used as a control. The resulting mixtures were used to infect a monolayer of Vero cells in 12-well plates with incubation for 60 min in an atmosphere of 5% CO_2_ at 37 °C. Following this, a 1 mL coating, consisting of medium 199 with Earle and Hanks salts (Chumakov FSC R&D IBP RAS (Institute of Poliomyelitis)) at a ratio of 2:1, 1.25% methylcellulose (Merck, Darmstadt, Germany), 0.1% gentamicin (Paneco), and 2% FBS, was added. Seven days post-infection, the coating was removed, cells were washed with PBS, fixed with 95% ethanol, and stained with 0.1% crystal violet dye (Sigma-Aldrich). The number of plaques for each dilution was then counted to calculate the neutralizing antibody titer. The calculation was made in logarithmic units using the following formula:

lg(nAB) = lg(A) + (f ∗ lg(d)),

where

A—dilution at which the number of plaques is less than 50% compared to control;

f—logarithm difference (interpolation coefficient), calculated as follows: f = (50% − M1)/(M2 − M1), where

M1—number of plaques is less than 50% compared to the control;

M2—number of plaques in the next dilution (plaques > 50%);

d—dilution factor.

The geometric mean titer of neutralizing antibodies (NAb GMT) was assessed in a neutralization reaction on Vero cell culture. Calculations were performed in logarithmic units using the Reed and Mench method. The titer value was determined based on the serum dilution at which the number of plaque-forming units decreased by more than 50% compared to the control. To refine the result, interpolation between neighboring dilution points was used. To minimize the limitations of this method (subjectivity in assessing the cytopathic effect, nonlinearity of the neutralization dependence), each sample was examined in three parallel settings, after which the average value was calculated. The minimum detection threshold for NAb was 1:10.

#### 2.7.7. Histological and Immunohistochemical Analyses

For histological evaluation, three time points were selected to capture the major phases of the antitumor response: day 7, corresponding to the early phase of virus replication and innate immune activation; day 14, corresponding to the point of maximum tumor volume before the onset of lysis; and days 21–30, corresponding to the active lysis phase in the combination therapy group and the expected peak of cytotoxic T-lymphocyte infiltration. At each time point, six mice from each experimental group were euthanized for tissue collection. The following groups were included: tumor-bearing untreated mice, tumor-bearing mice treated with ruxolitinib alone, tumor-bearing mice treated with YFV 17D alone (monotherapy), and tumor-bearing mice treated with the combination of YFV 17D and ruxolitinib. At necropsy, the primary tumor, the nearest draining lymph node, and the spleen were collected from each animal. Tissues were fixed in 10% neutral-buffered formalin (pH 6.8–7.2), dehydrated through a graded ethanol series, cleared, and embedded in paraffin according to standard histological procedures. Paraffin sections 3 μm thick were prepared for subsequent staining. For routine histological examination, sections were stained with hematoxylin and eosin (H&E).

Immunohistochemical staining was performed using a standard automated protocol on a Ventana Benchmark XT staining platform (Ventana Medical Systems, Tucson, AZ, USA) with the ultraView Universal DAB Detection Kit (Ventana). The following primary antibodies were used: anti-CD3 (Abcam (Cambridge, UK), cat. no. ab135372), anti-CD4 (Abcam, cat. no. ab237722), and anti-CD8 (Abcam, cat. no. ab217344). Immunostaining was performed for all treatment and control groups at each time point. Quantitative analysis of immunohistochemical staining was carried out using QuPath v.0.7.0 software. Positive cells were quantified as the number of immunoreactive cells per 1 mm^2^ of tissue area. For each marker, cell density was assessed in representative tumor sections using the same analysis settings across all groups to ensure comparability.

### 2.8. Statistical Analysis

Statistical analysis was performed using GraphPad Prism 8.2 (GraphPad Software, San Diego, CA, USA). Data are presented as mean ± standard error of the mean (SEM). For xCELLigence real-time cell proliferation assays, the curves represent the mean cell index of 8 technical replicates (wells) per condition. No formal statistical comparison between the cell groups was performed for xCELLigence assays due to the absence of independent biological replicates. Virus titer was measured in three independent biological replicates (n = 3). The data were obtained by plaque assay, and the values (PFU/mL) were log_10_-transformed prior to statistical analysis to account for the log-normal distribution of plaque assay data. Each biological replicate included all treatment conditions in duplicate (two technical replicates per condition in 12-well plates). The group value represents the mean of these two technical replicates. Comparisons across therapy and control cell groups were performed using one-way ANOVA with Dunnett’s multiple comparisons test. A *p*-value of less than 0.05 was considered statistically significant. For in vivo experiments, tumor growth curves were plotted for individual animals (n = 8) for each group. Overall survival was evaluated using the Kaplan–Meier method and compared between groups with the log-rank (Mantel–Cox) test. For the statistical analysis of the IHC and neutralizing antibody assay data, we first assessed the normality of distributions and then performed one-way ANOVA followed by Dunnett’s multiple comparisons test. Significance levels are indicated in figures as follows: * *p* < 0.05; ** *p* < 0.01; *** *p* < 0.001; **** *p* < 0.0001; ns—not significant.

## 3. Results

### 3.1. Heterogeneous Response of PDAC Cell Lines to YFV 17D and Ruxolitinib Combination Therapy

To test whether JAK1/2 kinase inhibition could sensitize pancreatic cancer cells to the oncolytic YFV 17D, we assessed cell viability in real time (xCELLigence) and virus replication in a panel of PDAC cell lines with different sensitivity to interferon, basal JAK/STAT axis activity, and genetic profiles (Table 1). Although all lines were treated with the YFV 17D and ruxolitinib combination under identical conditions, the lines’ responses were heterogeneous. This indicates that disruption of the antiviral interferon barrier in tumor cells does not determine a universal response to oncolytic virus therapy.

In our study, we identified three main patterns of response to the combination therapy: a synergistic effect (PAN02, PANC-1 cells), the lack of synergism (MiaPaCa-2 cells), and an increase in replicative activity of YFV 17D without a cytopathic effect (Capan-2, AsPC-1, and BxPC-3 cells). This distribution is likely due not only to the presence of an antiviral interferon barrier but also to its initial state, as well as the relationship between JAK/STAT-dependent signals and cell death mechanisms in a particular tumor line. As a result, some cells became significantly more sensitive to the virus after JAK/STAT blockade, while in other lines the same effect allowed the virus to replicate more efficiently but simultaneously decreased the cells’ ability to undergo apoptosis.

#### 3.1.1. Pattern 1: Synergistic Enhancement of Oncolytic Effect in PAN02 and PANC-1

**Figure 2:**
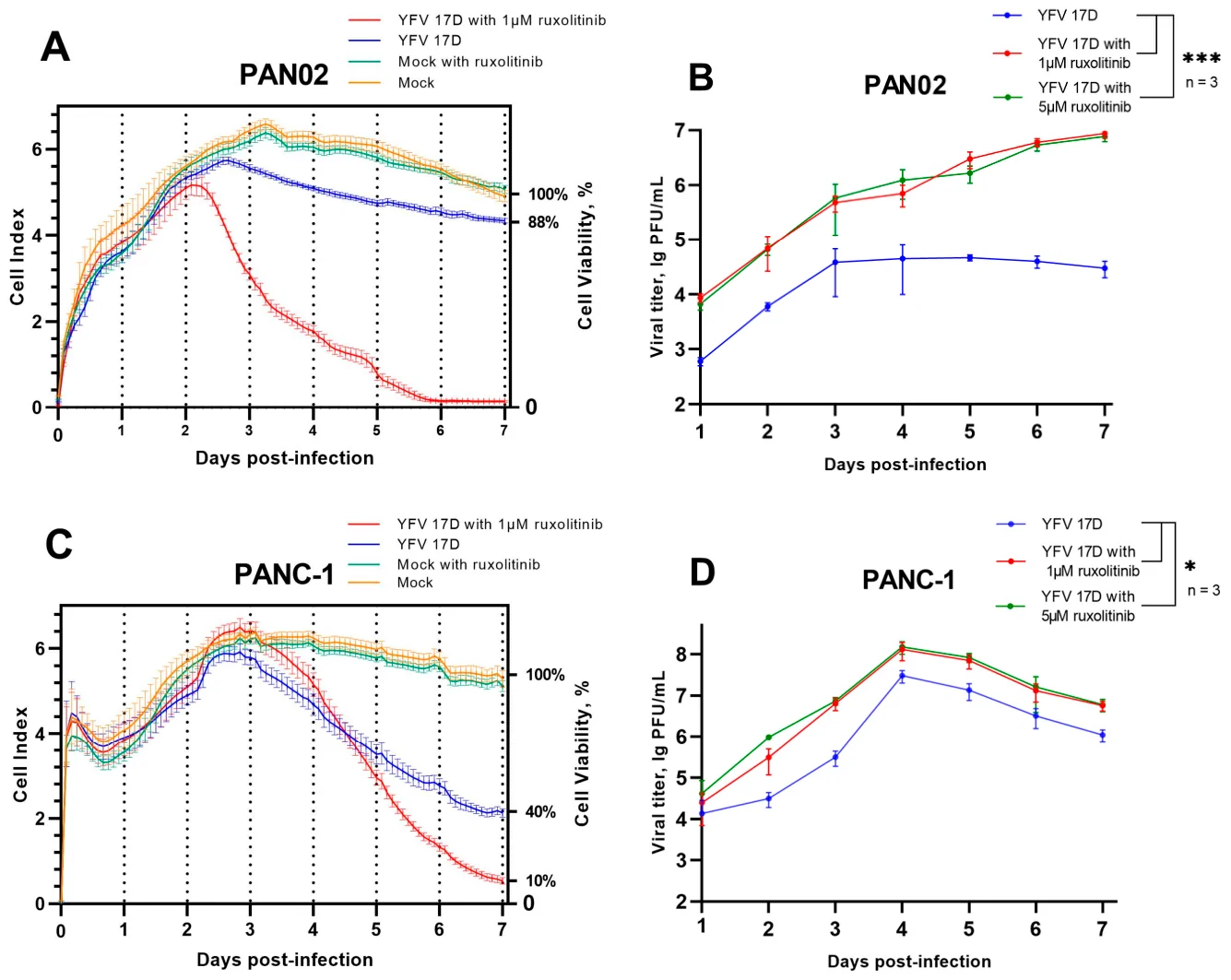
(**A**) Proliferation of PAN02 cells following mock treatment, treatment with 1 μM ruxolitinib, infection with YFV 17D (MOI = 1), or combined ruxolitinib and YFV 17D (MOI = 1) treatment. (**B**) YFV 17D titers in PAN02 cultures, PFU/mL (n = 3; *** *p* < 0.001). (**C**) Proliferation of PANC-1 cells following mock treatment, treatment with 1 μM ruxolitinib, infection with YFV 17D (MOI = 1), or combined ruxolitinib and YFV 17D (MOI = 1) treatment. (**D**) YFV 17D titers in PANC-1 cultures, PFU/mL (n = 3; * *p* < 0.05).

The most pronounced synergistic effect of combination therapy was found in PAN02 cells. Earlier, we tested ruxolitinib concentrations ranging from 0.1 to 5 µM and YFV 17D MOI ranging from 10^−4^ to 1 and determined the effective concentrations of ruxolitinib and YFV 17D in PDAC cell lines to be 1 µM and MOI 1, respectively. When treated with ruxolitinib at a concentration of 1 µM and infected with YFV 17D at MOI 1, almost complete cell death was observed by day 6, whereas treatment with 17D alone (monotherapy) caused the death of no more than 12% of cells (Figure 2A). At the same time, the virus titer with combination therapy increased by approximately two orders of magnitude by day 7, whereas with monotherapy, it decreased by approximately one order of magnitude (Figure 2B). Thus, for PAN02, the combination of YFV 17D with ruxolitinib was accompanied by a simultaneous increase in both the virus’ replicative activity and its cytopathic effect. We propose that the augmented oncolytic activity observed in PAN02 cells is primarily due to species-specific NS5-STAT2 interactions. PAN02 cells express murine STAT2 (mSTAT2), to which the YFV NS5 protein binds inefficiently, in contrast to human STAT2 [9]. Consequently, in the PAN02 line, the virus alone is apparently unable to completely suppress the interferon barrier, and the JAK1/2 blockade by ruxolotinib is critically important for the development of the virus’ oncolytic potential.

A similar, though less pronounced, effect was observed in PANC-1 cells. The addition of 1 µM of ruxolitinib and YFV 17D at an MOI = 1 led to almost complete cell death by day 7, while the addition of YFV 17D alone caused the cytopathic effect that did not exceed 60% (Figure 2C). In the case of the YFV17D+ruxolitinib combination, the virus titer in the supernatant was also approximately one order of magnitude higher and reached a maximum by day 4 (Figure 2D). For PANC-1, the synergism probably indicates the presence of a partially functional but heterogeneous interferon response. According to the published research data, this cell line is characterized by heterogeneity in IFNAR1/IFNAR2 expression and the presence of subpopulations with different levels of sensitivity to interferon, which suggests the existence of IFNAR-upregulated and IFNAR-downregulated clones [11]. Therefore, the virus presumably mostly affects the clones with impaired interferon response (IFNAR-downregulated), whereas the addition of ruxolitinib eliminates the JAK/STAT-mediated antiviral barrier in IFNAR-upregulated cells and allows the virus to replicate more efficiently.

#### 3.1.2. Pattern 2: Lack of Synergism in MiaPaCa-2 Cells

The MiaPaCa-2 cell line originates from a primary pancreatic tumor and harbors *KRAS* and *TP53* mutations but has significantly lower JAK/STAT and ISG basal activity levels compared to the Capan-2 and AsPC-1 cell lines [20]. MiaPaCa-2 is a classical PDAC subtype (in contrast to the basal-like subtype of Capan-2 and AsPC-1 cell lines) and is more differentiated and less aggressive. Its specific combination of the G12C KRAS mutation (associated with decreased JAK/STAT pathway activity compared to G12D or G12V mutations) and the R248W p53 mutation may contribute to its impaired basal interferon response.

**Figure 3:**
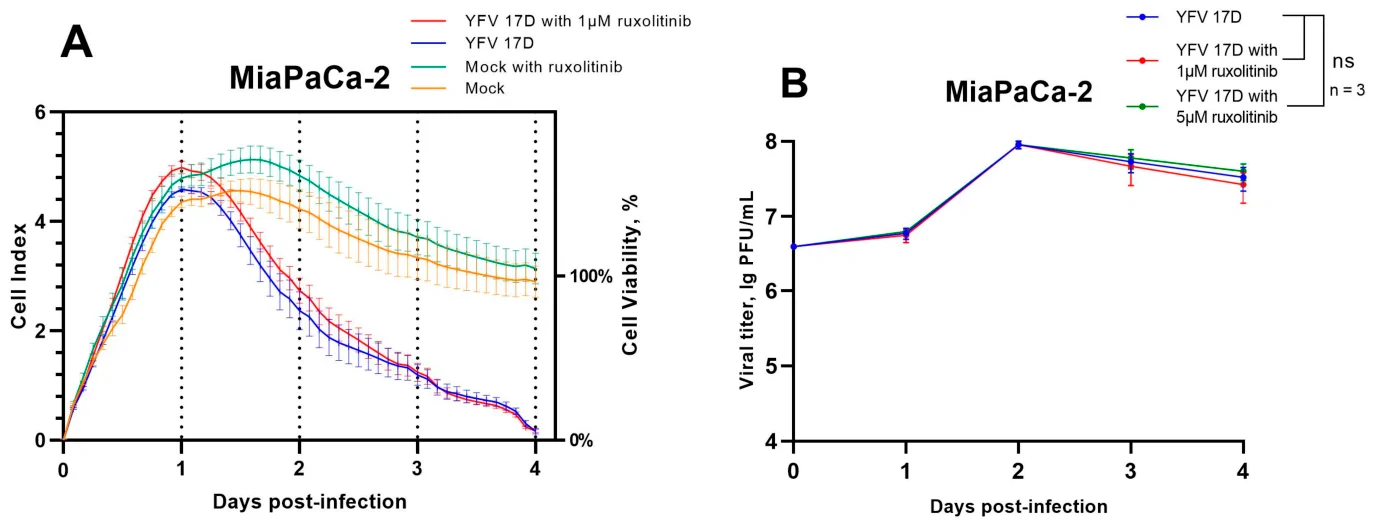
(**A**) Proliferation of MiaPaCa-2 cells following mock treatment, treatment with 1 μM ruxolitinib, infection with YFV 17D (MOI = 1), or combined ruxolitinib and YFV 17D (MOI = 1) treatment. (**B**) YFV 17D titers in MiaPaCa-2 cells, PFU/mL (n = 3; ns—not significant).

The addition of ruxolitinib did not enhance the cytopathic effect of 17D on MiaPaCa-2 cells. Combined treatment with ruxolitinib (1 μM) and YFV 17D (MOI = 1) resulted in complete tumor cell death by day 4 post-treatment, similar to that seen in cells infected with 17D alone (Figure 3A). Virus titers in cells with combination therapy were similar to those with monotherapy and peaked at day 2 and declined thereafter.

The lack of virus-induced CPE enhancement in response to treatment with ruxolitinib in MiaPaCa-2 cells is associated with impaired interferon response, which is consistent with the established literature [22]. The cells demonstrate the lowest basal expression levels of interferon signaling components (STAT1, STAT2, and IRF-9) among the studied PDAC lines and virtually undetectable basal ISG expression. Functionally, this is equivalent to the absence of the interferon barrier. Therefore, this allows YFV 17D to replicate in MiaPaCa-2 cells with no restrictions: in our experiment, its titer reached 10^8^ PFU/mL regardless of ruxolitinib presence (Figure 3B). By day 4, we observed complete YFV 17D-induced cell lysis, regardless of JAK/STAT inhibition (Figure 3A). Thus, JAK/STAT inhibition does not enhance YFV 17D replication due to the absence of functional interferon signaling pathways, rendering the use of ruxolitinib redundant in the case of MiaPaCa-2 cells.

These results provide an important negative control, demonstrating that the augmenting effect of ruxolitinib on YFV 17D-based virotherapy depends on interferon response viability in different PDAC cell types. In cell lines with dysfunctional interferon signaling, JAK/STAT inhibition is redundant. We believe that this finding may be of clinical practice importance, as PDAC tumors are heterogeneous in their interferon signaling functionality, and patients with tumors phenotypically resembling MiaPaCa-2 (with impaired interferon signaling) would receive minimal benefit from ruxolitinib-enhanced virotherapy.

#### 3.1.3. Pattern 3: Enhanced Viral Replication with No Cytopathic Effect in Capan-2, AsPC-1, and BxPC-3 Cells

Despite the differences in their mutation profiles, cell lines Capan-2 (KRAS G12V, p53 WT), AsPC-1 (KRAS G12D, p53-null), and BxPC-3 (KRAS WT, p53 Y220C, non-functional SMAD4) demonstrated surprisingly similar responses to both YFV 17D monotherapy and its combination with ruxolitinib. All three lines are described as having high basal activity levels of the JAK/STAT pathway and post-receptor interferon signaling, as well as increased resistance to oncolytic viruses [20,22].

**Figure 4:**
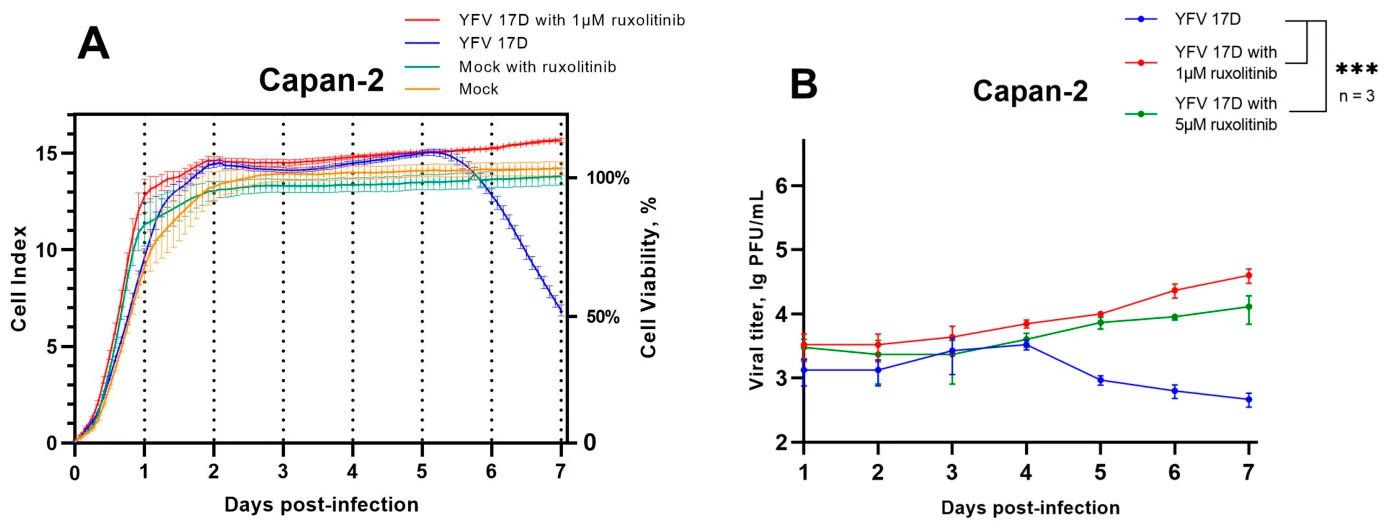

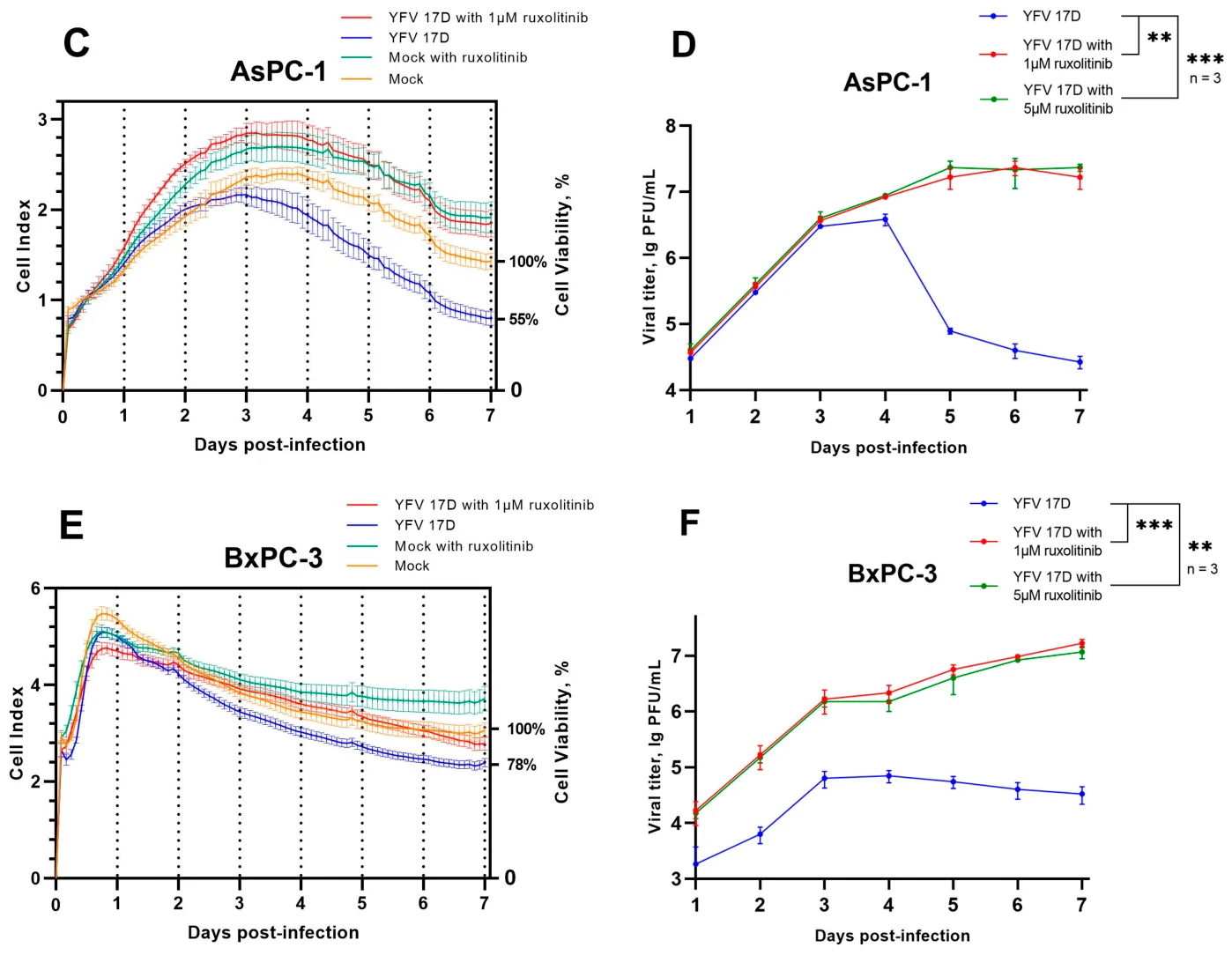
(**A**) Proliferation of Capan-2 cells following mock treatment, treatment with 1 μM ruxolitinib, infection with YFV 17D (MOI = 1), or combined ruxolitinib and YFV 17D (MOI = 1) treatment. (**B**) YFV 17D titers in Capan-2 cultures, PFU/mL (n = 3; *** *p* < 0.001). (**C**) Proliferation of AsPC-1 cells following mock treatment, treatment with 1 μM ruxolitinib, infection with YFV 17D (MOI = 1), or combined ruxolitinib and YFV 17D (MOI = 1) treatment. (**D**) YFV 17D titers in AsPC-1 cultures, PFU/mL (n = 3; ** *p* < 0.01; *** *p* < 0.001). (**E**) Proliferation of BxPC-3 cells following mock treatment, treatment with 1 μM ruxolitinib, infection with YFV 17D (MOI = 1), or combined ruxolitinib and YFV 17D (MOI = 1) treatment. (**F**) YFV 17D titers in BxPC-3 cultures, PFU/mL (n = 3; ** *p* < 0.01; *** *p* < 0.001).

The addition of 1 µM of ruxolitinib to Capan-2 cells did not enhance the YFV 17D-induced cytopathic effect, which was inoculated at an MOI = 1. Remarkably, monotherapy with YFV 17D alone led to the death of approximately 50% of cells by day 7 (Figure 4A), whereas in the case of the combination therapy, cell viability was at the same level as in the control group cells. However, the addition of ruxolitinib significantly increased the YFV 17D replicative activity in Capan-2 cells, while in the absence of ruxolitinib, the virus titer rapidly decreased by day 7 (Figure 4B). A similar effect was observed in AsPC-1 cells: monotherapy with YFV 17D resulted in the death of approximately 45% of cells by day 7, while the addition of ruxolitinib reduced the YFV 17D-induced cytopathic effect (Figure 4C) but increased the virus titers from 10^5^ to 10^7^ PFU/mL over the same period (Figure 4D). In the BxPC-3 cells, which have the highest level of post-receptor IFN signaling activity among the studied PDAC lines, YFV 17D alone did not cause a significant cytopathic effect (we observed the death of approximately 22% of cells by day 7) (Figure 4E). The addition of ruxolitinib also did not enhance the cytopathic effect but caused an increase in the virus titers from ~10^4^ to ~10^7^ PFU/mL (Figure 4F).

**Figure 5:**
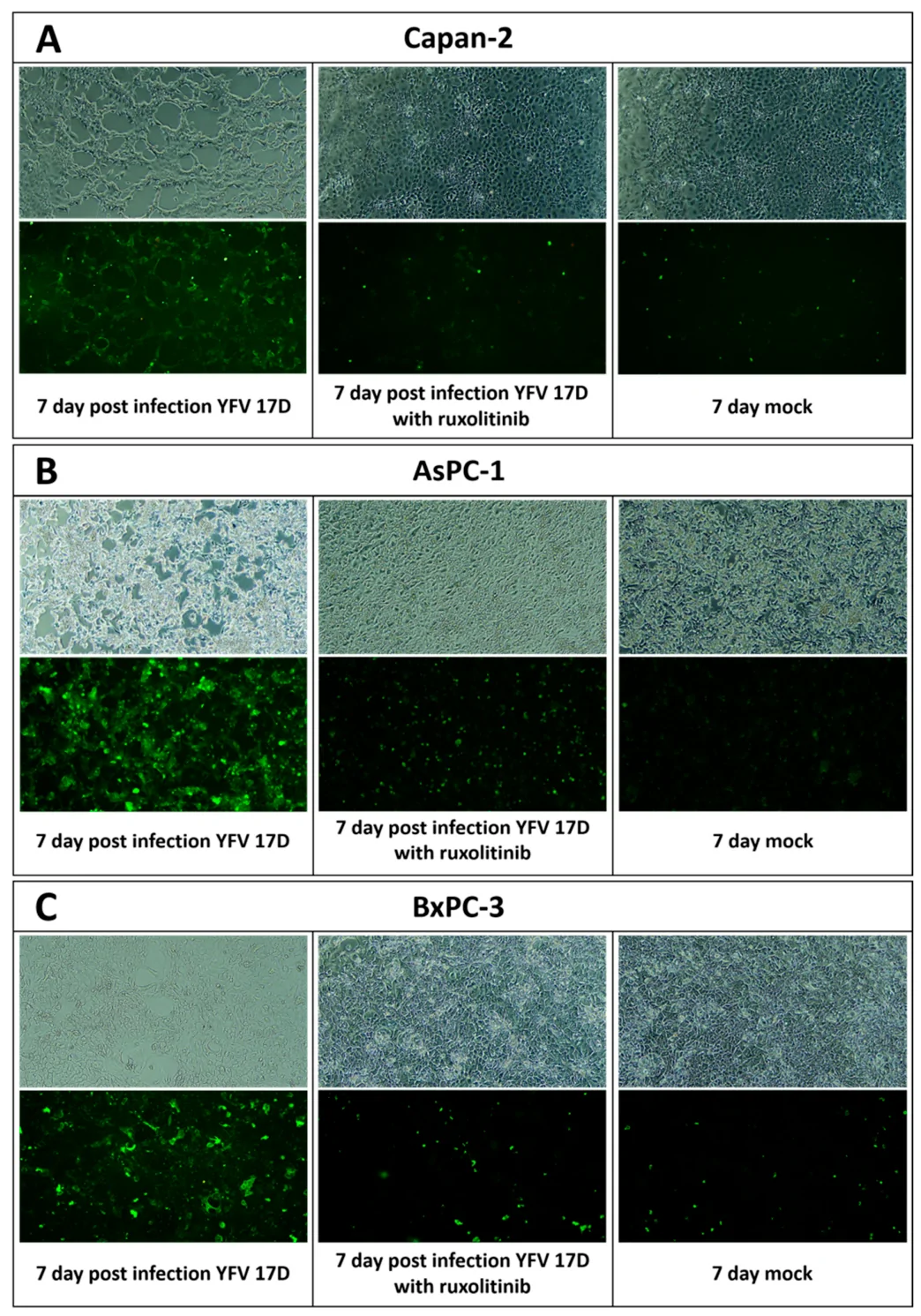
Annexin V-FITC fluorescence microscopy shows YFV 17D-induced apoptosis in (**A**) Capan-2, (**B**) AsPC-1, and (**C**) BxPC-3 cells and its suppression by ruxolitinib. Cells were infected with YFV 17D with or without the addition of ruxolitinib and stained with Annexin V-FITC on day 7 post-infection; representative bright-field and corresponding FITC fluorescence images (×100 magnification) are shown for mock, YFV 17D alone, and YFV 17D with ruxolitinib conditions.

After analyzing the data obtained, we hypothesized that YFV 17D monotherapy in Capan-2, AsPC-1, and BxPC-3 cells (in all three, the virus titers decreased) leads to apoptotic cell death. To directly confirm the type of cell death observed in these three cell lines during YFV 17D monotherapy, we performed repeated experiments and stained the cells with Annexin V–FITC, followed by fluorescence microscopy on day 7 after the therapy (Figure 5). In cells from the YFV 17D monotherapy group, Annexin V-positive cells were found in all three cell lines, indicating phosphatidylserine externalization and apoptosis, which is consistent with the cytopathic effect measured by impedance analysis. In contrast, in the group receiving combined YFV 17D and ruxolitinib therapy, comparatively fewer Annexin V-positive cells were observed, despite significantly higher virus titers in the culture medium. In the control group of cells that did not receive the treatment, no apoptotic signal was observed. The intensity of the fluorescent signal in Capan-2 (Figure 5A) cells during YFV 17D monotherapy was lower than in AsPC-1 (Figure 5B) and BxPC-3 (Figure 5C) cells. These results confirm that the type of cell death we observed with YFV 17D monotherapy in Capan-2, AsPC-1, and BxPC-3 cells was apoptotic and that ruxolitinib specifically suppresses apoptotic cell death that otherwise would be induced as a response to the YFV 17D infection.

Taken together, these data indicate that in Capan-2, AsPC-1, and BxPC-3 cells, ruxolitinib enhances YFV 17D replication while limiting the cytopathic effect: inhibition of JAK1/2 kinases weakens cells’ interferon-mediated antiviral response along with proapoptotic signaling necessary for the virus-induced cell death. The similarity of the responses in these cell lines, despite the differences in their *KRAS* and *TP53* mutation profiles, suggests that the main factor determining the outcome of oncolytic virotherapy in this subgroup of pancreatic cancers is basal activity levels of the JAK/STAT and interferon signaling pathways, rather than individual oncogenic mutations per se.

### 3.2. Controlled Use of Ruxolitinib Enhances the Oncolytic Activity of YFV 17D in the PAN02 Syngeneic Model

The data obtained on the panel of PDAC cell lines and presented in the previous section indicate that the combination of YFV 17D with ruxolitinib provides a significant enhancement of virus replication and, in some cases, the cytopathic effect in a number of pancreatic cancer cell lines. In order to verify the antitumor efficacy of this combination in vivo and analyze its immunological mechanisms, we conducted an experiment on a syngeneic PDAC PAN02 mouse model [28]. This model reproduces the key immune microenvironment features of this disease in humans. It is widely used for preclinical evaluation of antitumor immunotherapy strategies: the intact host immune system provides an opportunity to study both direct oncolytic mechanisms of the virus and the contribution of the adaptive immune response to the antitumor effect [29].

PAN02 cells (no more than 2 × 10^6^ cells in a volume of 100 µL) were injected subcutaneously into the lateral surface of the left thigh of female C57BL/6 mice. After the tumor reached a diameter of 5 mm, the animals were randomized into four groups. The control group received intracellular administration of a sterile phosphate-salt buffer solution. The ruxolitinib control group received intraperitoneal administration of ruxolitinib and intratumoral administration of a sterile phosphate-salt buffer solution. The YFV 17D monotherapy group received a single intratumoral administration of YFV 17D (10^5^ PFU in a 100 µL volume). The combination therapy group received a single intratumoral administration of YFV 17D (10^5^ PFU in a 100 µL volume). Ruxolitinib at a concentration of 10 mg/mL was administered intraperitoneally 24 and 3 h prior to the administration of YFV 17D, then 24 and 48 h after the introduction of the virus. The tumor size was assessed daily with a caliper. The humane endpoint was a tumor size of 500 mm^3^ or a deterioration of the animal’s condition. The dose of ruxolitinib 10 mg/mL intraperitoneally corresponds to the range used in previously published preclinical studies of the combination of JAK inhibitors with oncolytic viruses in PDAC models [30].

**Figure 6:**
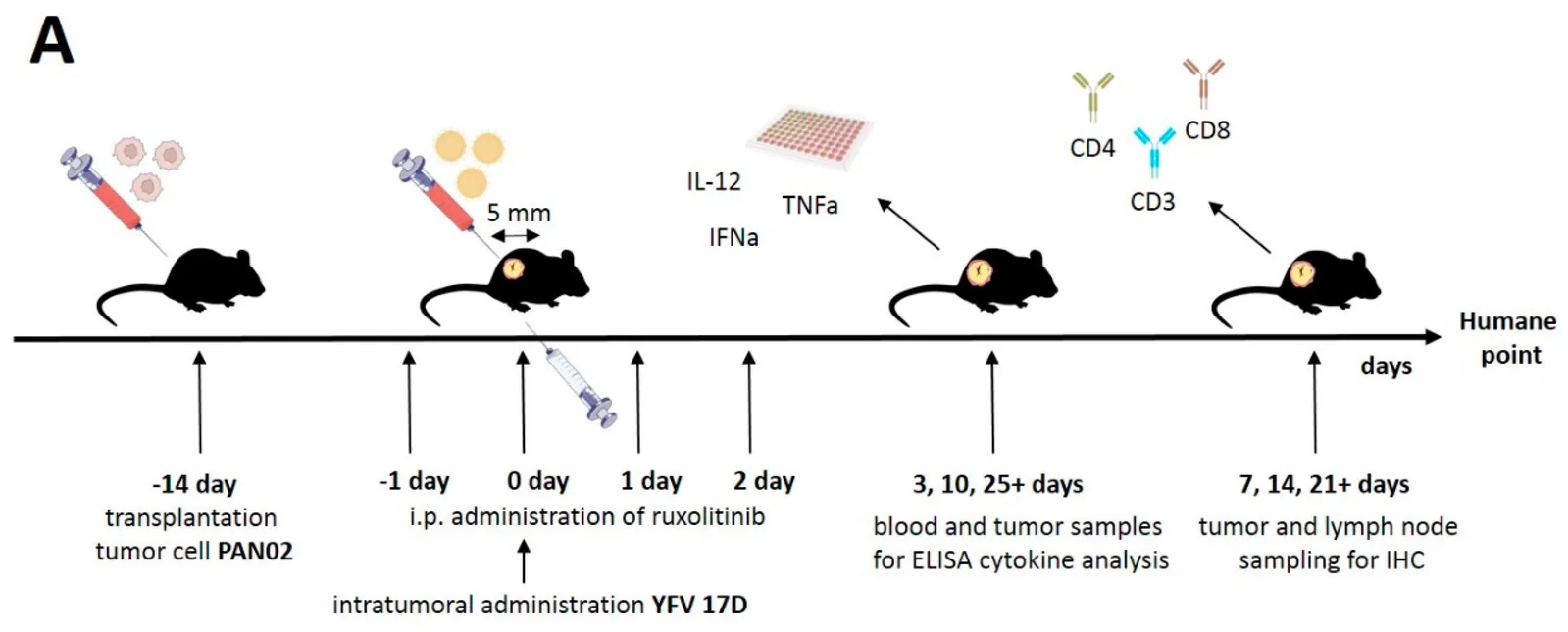

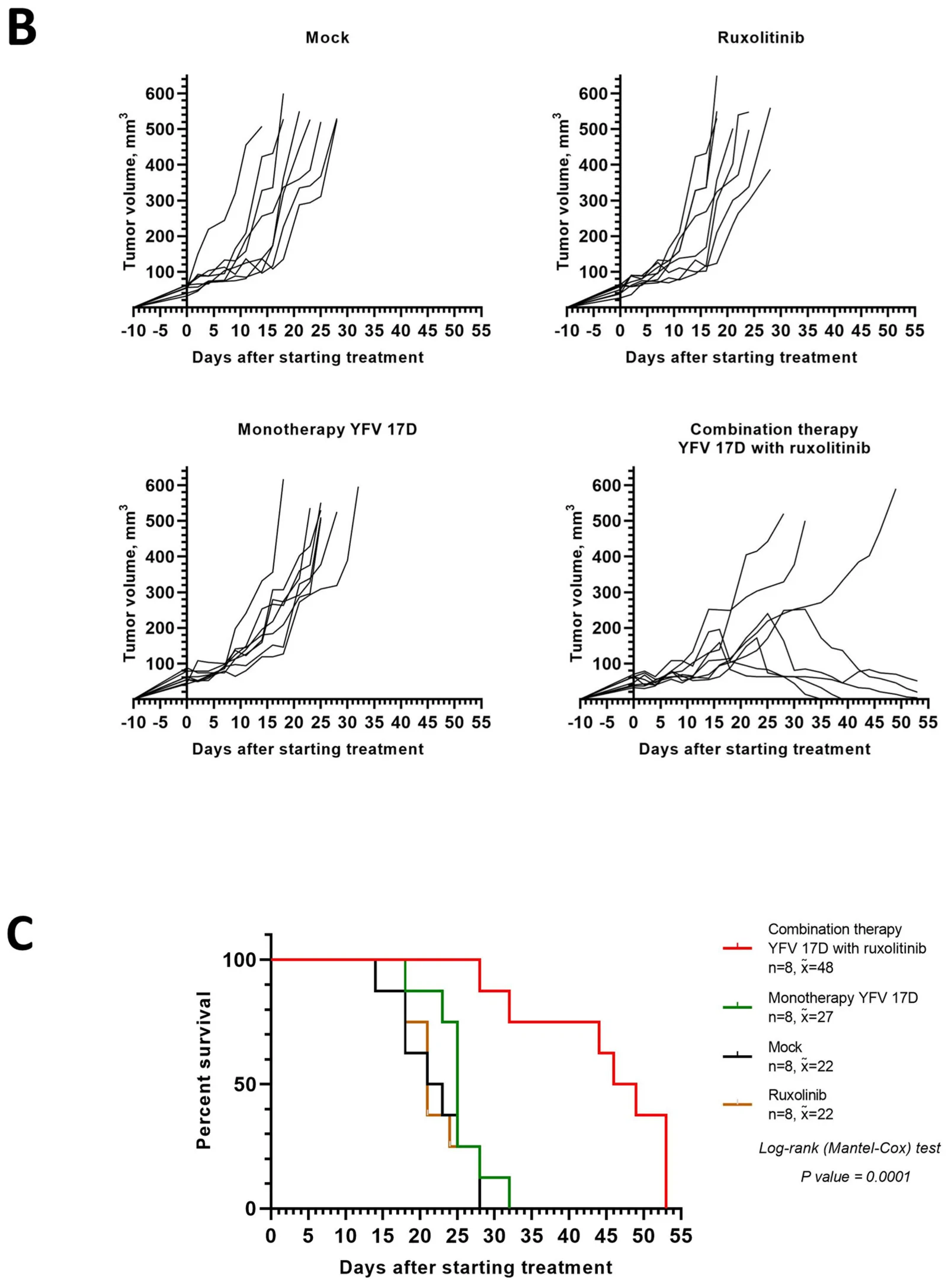
(**A**) PAN02 syngeneic model experiment scheme; (**B**) Graphs of the tumor growth dynamics in the YFV 17D monotherapy group, the YFV 17D combination therapy group, and the control group (Mock and Ruxolitinib); (**C**) Survival analysis using the Kaplan–Meier method in the YFV 17D monotherapy group, YFV 17D combination therapy group, and the control group (Mock and Ruxolitinib). Mantel–Cox log-rank test, *p* = 0.0001.

Analysis of the tumor growth dynamics showed that in the control group, tumors exhibited stable growth (Figure 6B). In the YFV 17D monotherapy group, a single intracellular administration of the virus resulted in only a moderate delay in tumor growth without evident regression, which corresponds to the data we previously obtained studying monotherapy with YFV 17D in this model.

In the combination therapy group, 5 out of 8 animals (62.5%) had partial or complete tumor lysis (Figure 6B). We observed a recurrent dynamic in the response pattern: the initial tumor growth was followed by tumor regression and, eventually, lysis. In a number of animals, the tumors with incomplete lysis continued to grow, and once more, tumor lysis followed. This could indicate the formation of a stable adaptive antitumor immune response.

Kaplan–Meier survival analysis demonstrated statistically significant differences between the groups (Mantel–Cox log-rank test, *p* = 0.0001) (Figure 6C). The median survival time to a humane endpoint in the combination therapy group was 48 days, whereas in the monotherapy group it was 27 days, and in the control group it was 22 days. Thus, combination therapy provided an increase in the median survival time to a humane endpoint by more than 2 times compared with the control group and by 1.8 times compared with the monotherapy group. Monotherapy with YFV 17D increased the median survival by 1.2 times compared with the control group.

We then conducted a PCR analysis of tumor homogenates collected on days 3 and 5 after administration of YFV 17D (n = 3 for each group). It did not reveal significant differences in the YFV 17D RNA levels between monotherapy and combination therapy groups (Table 2). Hence, the enhancing properties of ruxolitinib in vivo cannot be attributed to a steady increase in YFV 17D intratumoral replication and an increase in its cytopathic effect, as we observed in vitro in PAN02 cells. Instead, these data suggest that the short-term inhibition of JAK1/2 kinases changes the early antiviral and inflammatory context in vivo, which contributes to the formation of a specific adaptive antitumor immune response.

To test the safety of the combination therapy, we PCR-analyzed the mice’s brain and spleen samples from the combination therapy group for the presence of the YFV 17D RNA. All the samples were negative for YFV 17D RNA (Table 2). We also performed a plaque assay on the Vero cell culture using these samples to further exclude any possibility of the presence of YFV 17D in them. It showed no plaques and, consequently, the absence of infectious viral particles in the samples. Taken together, these results indicate the absence of neuro- and systemic dissemination of YFV 17D used for intratumoral administration in combination with ruxolitinib, which confirms the safety of the combined therapy.

### 3.3. Combination Therapy Transiently Suppresses Early IFNα Expression and Promotes a Later IL-12-Associated Immune Phase

To assess the local immune response in the tumor microenvironment, we measured IFN-α, TNF-α, and IL-12 levels in tumor tissue homogenates derived from animals in all three groups on the 3rd, 10th, and 25th days after the start of therapy (n = 4 in each group at each time point). Remarkably, the levels of all three cytokines in the serum were below the sensitivity thresholds of the ELISA kits used. This indicates the absence of a systemic cytokine release and the exclusively intratumoral nature of immunological changes induced by the combination therapy.

**Figure 7:**
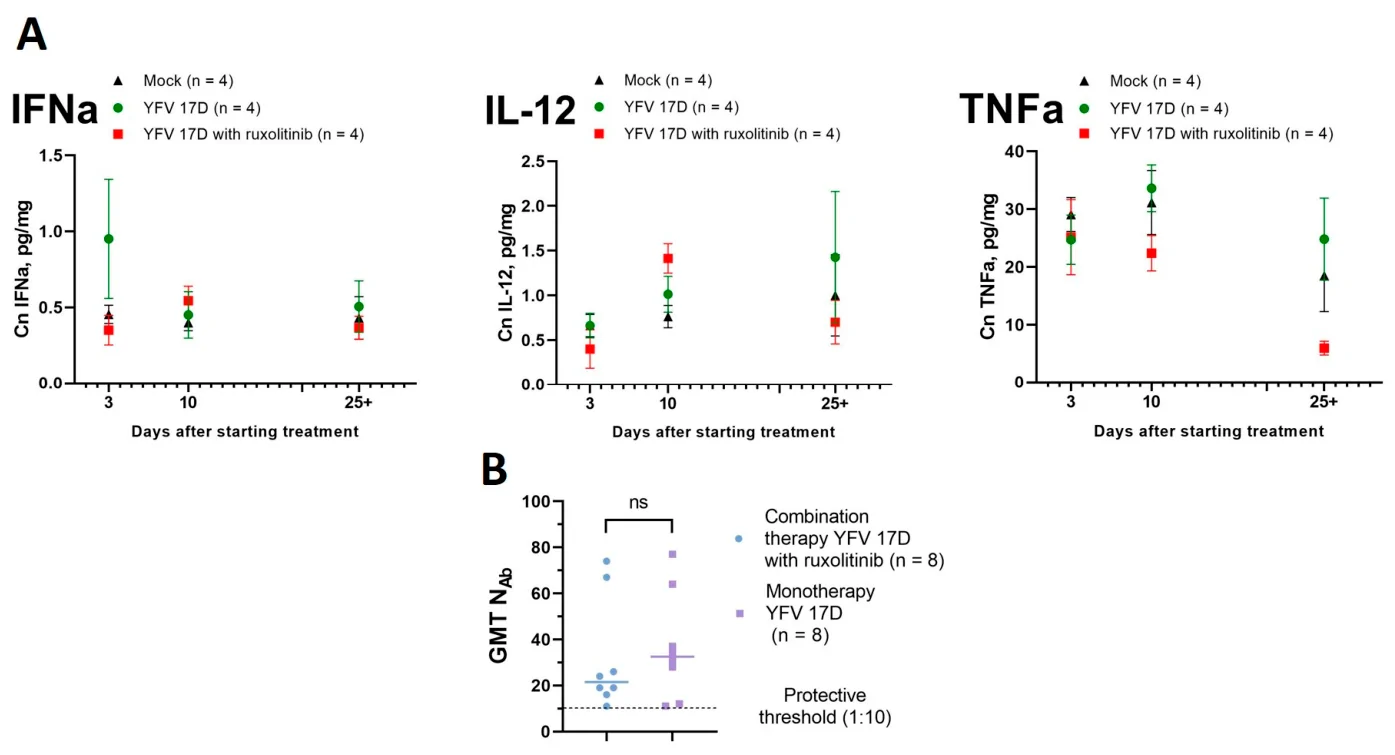
(**A**) Intratumoral cytokine levels after YFV 17D and combination therapy: levels IFNa (Cn IFNa, pg/mg protein, n = 4); levels TNFa (Cn TNFa, pg/mg protein, n = 4); levels IL-12 (Cn IL-12, pg/mg protein, n = 4) Cytokine levels were quantified using commercial ELISA kits specific for murine IFN-α, TNF-α, and IL-12 (Cloud-Clone Corp, cat. no. IEA033Mu, SEA133Mu, SEA058Mu) according to the manufacturers’ protocols and normalized to total protein content. Cytokine concentrations are reported as pg per mg of total protein—Cn, pg/mg. (**B**) Geometric mean titers of neutralizing antibodies to YFV 17D after intratumoral therapy (GMT N_Ab_, n = 8; ns—not significant). The geometric mean titer of neutralizing antibodies (NAb GMT) was assessed in a neutralization reaction on Vero cell culture. Calculations were performed in logarithmic units using the Reed and Mench method. The titer value was determined based on the serum dilution at which the number of plaque-forming units decreased by more than 50% compared to the control. To refine the result, interpolation between neighboring dilution points was used. To minimize the limitations of this method (subjectivity in assessing the cytopathic effect, nonlinearity of the neutralization dependence), each sample was examined in three parallel settings, after which the average value was calculated. The minimum detection threshold for NAb was 1:10.

On day 3 after the start of therapy (the period of primary virus replication), we observed a significant increase in IFN-α levels (~0.97 pg/mg) in the tumor homogenates derived from the YFV 17D monotherapy group. This exceeded IFN-α levels in the control group (~0.45 pg/mg) approximately 2 times (Figure 7A). In contrast, IFN-α levels in the combination therapy group were noticeably lower (~0.35 pg/mg), comparable to or slightly lower than the control group ranges. On the 10th and after the 25th day, IFN-α levels in all three groups evened out and ranged from 0.4 to 0.55 pg/mg without statistically significant differences between the groups. Suppression of the early IFN-α response in the combination therapy group is an expected consequence of JAK1/2-dependent signaling inhibition by ruxolitinib: the blockade of STAT1/STAT2 phosphorylation downregulates ISG, and, consequently, IFN-I expression in both tumor and tumor-infiltrating immune cells [31]. This suppression is temporary and local: it does not affect the blood nor does it persist for long periods of time, which is consistent with the short-term regimen of ruxolitinib used in this experiment.

TNF-α levels in all three groups on day 3 were comparable (~25–29 pg/mg) (Figure 7A). On day 10, TNF-α levels in the YFV 17D monotherapy group increased (~33 pg/mg) and decreased in the combination therapy group (~22 pg/mg). The control group TNF-α levels remained stable at ~30 pg/mg. The most significant differences were observed after day 25: in the combination therapy group, TNF-α levels rapidly decreased to ~6 pg/mg, whereas in the YFV 17D monotherapy group they remained at ~25 pg/mg, and in the control group they were ~18 pg/mg. We suggest that this rapid TNF-α decrease in the combination therapy group by day 25 may be a consequence of the tumor mass reduction due to the antitumor immune response (tumor regression was observed in 5 out of 8 animals in this group). Tumor regression is followed by a decrease in tumor-associated inflammation and, therefore, a decrease in TNF-α production in the residual tumor tissue.

The IL-12 dynamics in tumor homogenates were remarkably different. On day 3, IL-12 levels in all groups were low and comparable (~0.4–0.65 pg/mg), with the combination therapy group having the lowest level (~0.4 pg/mg) (Figure 7A). On day 10, the combination therapy group showed the most pronounced increase (~1.45 pg/mg) compared with the YFV 17D monotherapy (~1.0 pg/mg) and control (~0.85 pg/mg) groups. After day 25, the dynamics changed: we observed a decrease to ~0.7 pg/mg in the combination therapy group and an increase to ~1.45 pg/mg in the YFV 17D monotherapy group. In the control group, the value was ~1.0 pg/mg. The peak level of IL-12 on day 10, observed in the combination therapy group, may indicate the activation of IL-12–producing cells such as dendritic cells and M1-type macrophages during the early phase of the immune response to the virus, which was preceded by the blockade of the JAK/STAT-mediated antiviral response by ruxolitinib [32,33]. IL-12 is a key cytokine that determines Th1 polarization and priming of the cytotoxic CD8+ T cell response. Its early intratumoral increase during combination therapy favors the formation of the adaptive antitumor response [34]. Its subsequent decrease by day 25 in the combination therapy group is, plausibly, a consequence of the acute phase of infection resolution. As the virus is eliminated, the immune response shifts from innate to adaptive, and the IL-12-dependent priming phase gives way to the phase of CD8+ T cell activity [35].

To assess the humoral antiviral response after the intratumoral administration of YFV 17D, we measured virus-neutralizing antibodies in mice from both the combination and monotherapy groups. Neutralizing antibodies play a crucial role in the formation of the specific adaptive immune response to the yellow fever virus. According to the published data, the protective threshold for the yellow fever virus in neutralization assays is the titer of approximately 1:10, depending on the test system used and the analysis endpoint [36]. We detected neutralizing antibodies to YFV 17D in both groups (Figure 7B). In all the mice, the values were within the range of the protective titer of neutralizing antibodies or above it, which indicates the formation of a specific humoral response to the virus. Comparative analysis showed no statistically significant differences in neutralizing antibody levels between the combination therapy and monotherapy groups. This indicates that ruxolitinib did not suppress the formation of the specific humoral antiviral response to YFV 17D. This observation is important because the JAK/STAT signaling pathway inhibition can alter the early interferon response and affect the virus’ interaction with tumor cells; however, according to the data obtained, it does not prevent the subsequent development of a virus-specific humoral immune response.

### 3.4. Morphological Findings

**Figure 8:**
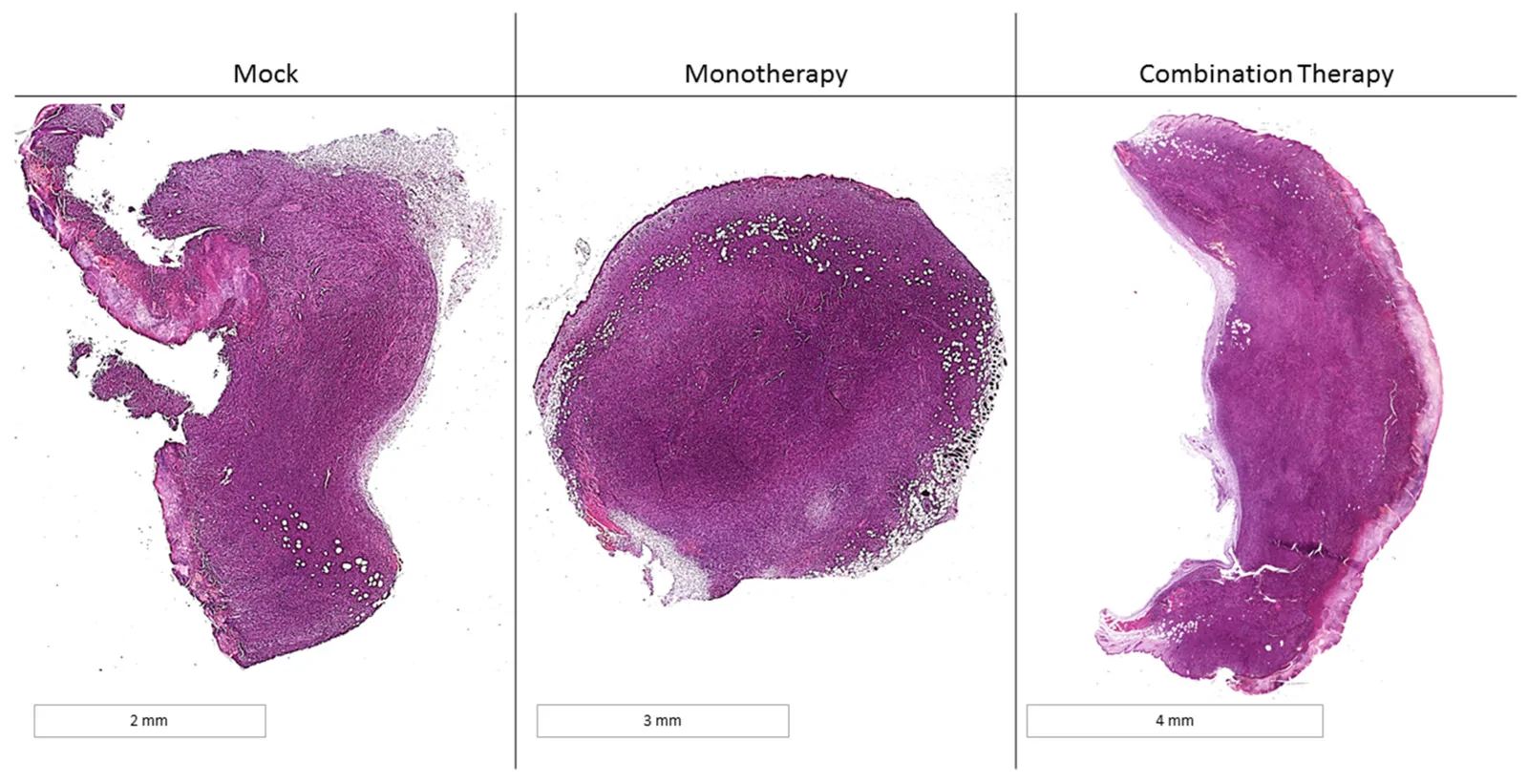
Representative histological images showing tumor borders and morphology after mono- and combination therapy on day 14.

**Figure 9:**
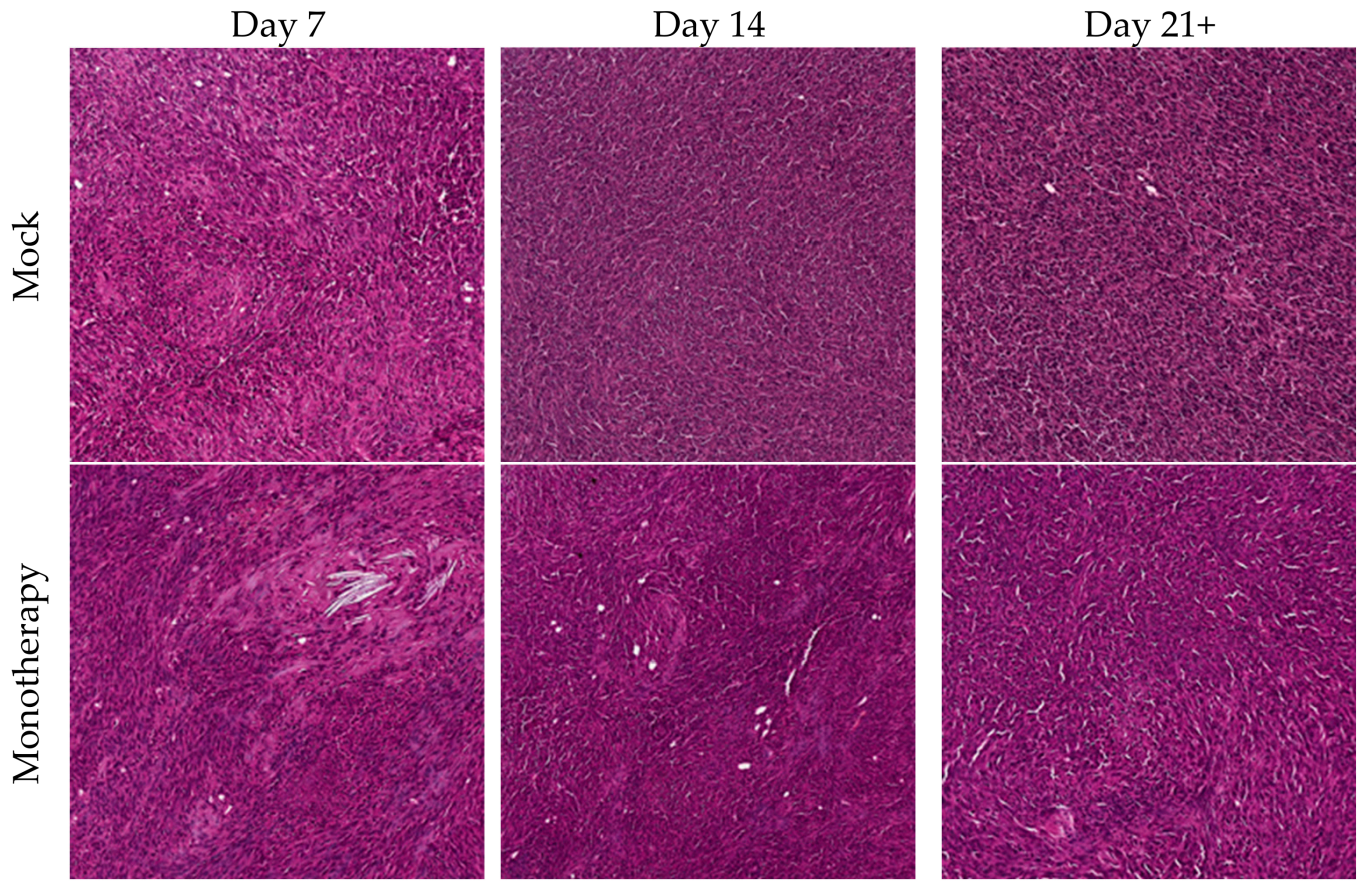

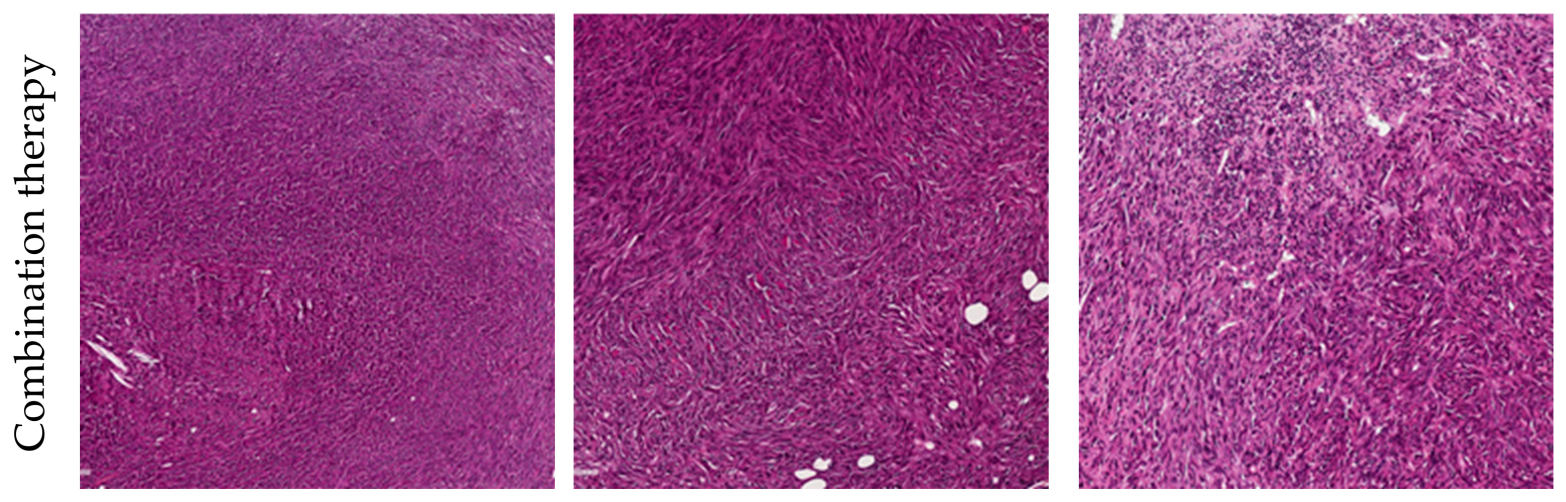
Histological changes in tumors over time in response to mono- and combination therapy (H&E staining, ×200; days 7, 14 and 21+).

Histological examination of tumors from animals in all groups confirmed the tumor type as pancreatic ductal adenocarcinoma (PDAC). Their histological architecture varied depending on the treatment methods and the tumor progression/regression dynamics. In the control group (mock), the tumors consisted of glandular-type cancer cells with multiple mitotic divisions concomitant with tumor growth. Remarkably, we observed no tumor invasion into the surrounding tissues in animals from the combination therapy group. However, we also observed occasional cells with apoptotic changes. In the monotherapy group, the number of dying cells in tumor tissue was moderately higher, and we observed foci of necrosis and a small number of diffusely scattered lymphocytes (Figure 8 and Figure 9).

The most distinct signs of tumor regression and destruction, such as apoptosis, necrosis, and lymphoid infiltration, were detected in samples from animals in the combination therapy group (Figure 9). Histological analysis of tumors in the combination therapy group revealed the loss of density and significant structural changes in tumor tissue.

### 3.5. Immunohistochemical Staining

**Figure 10:**
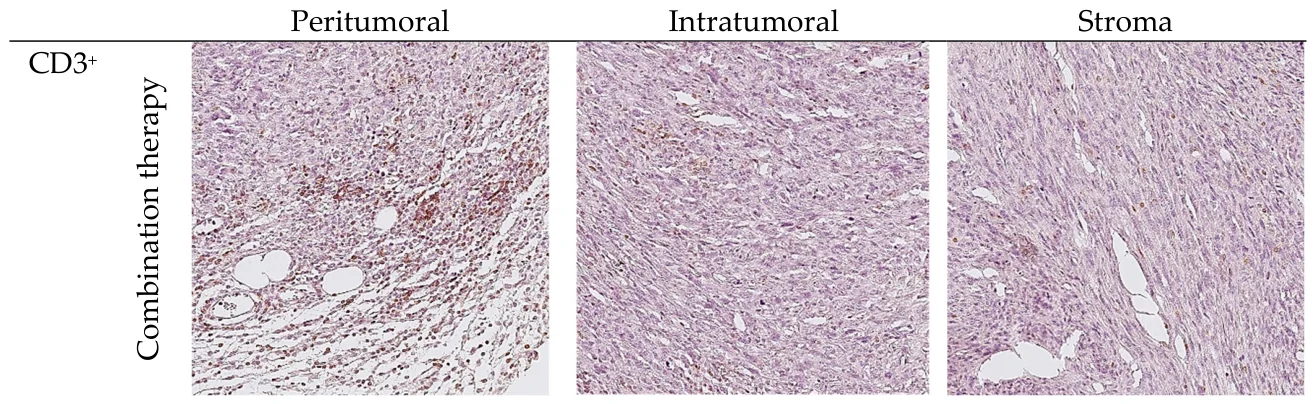

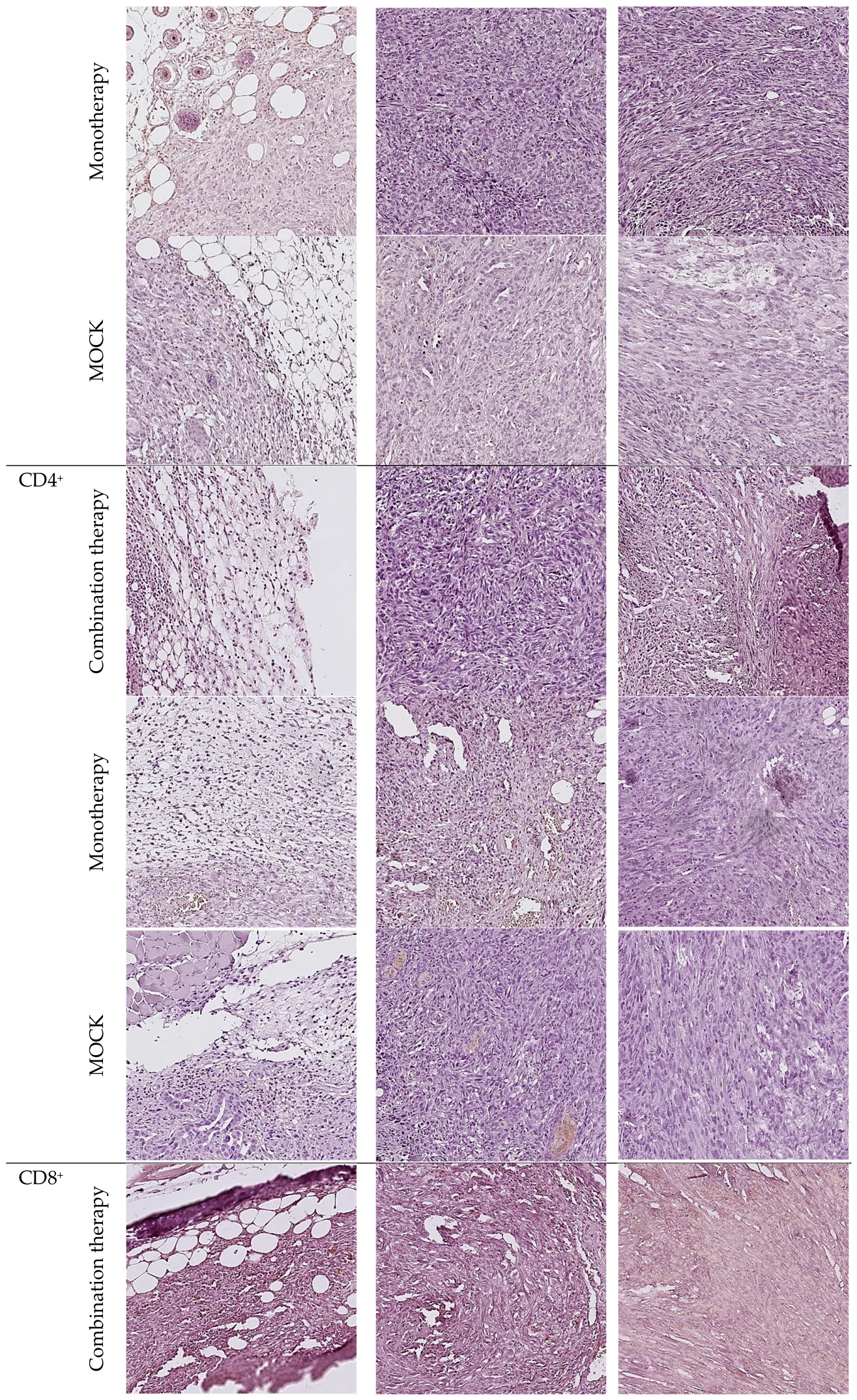

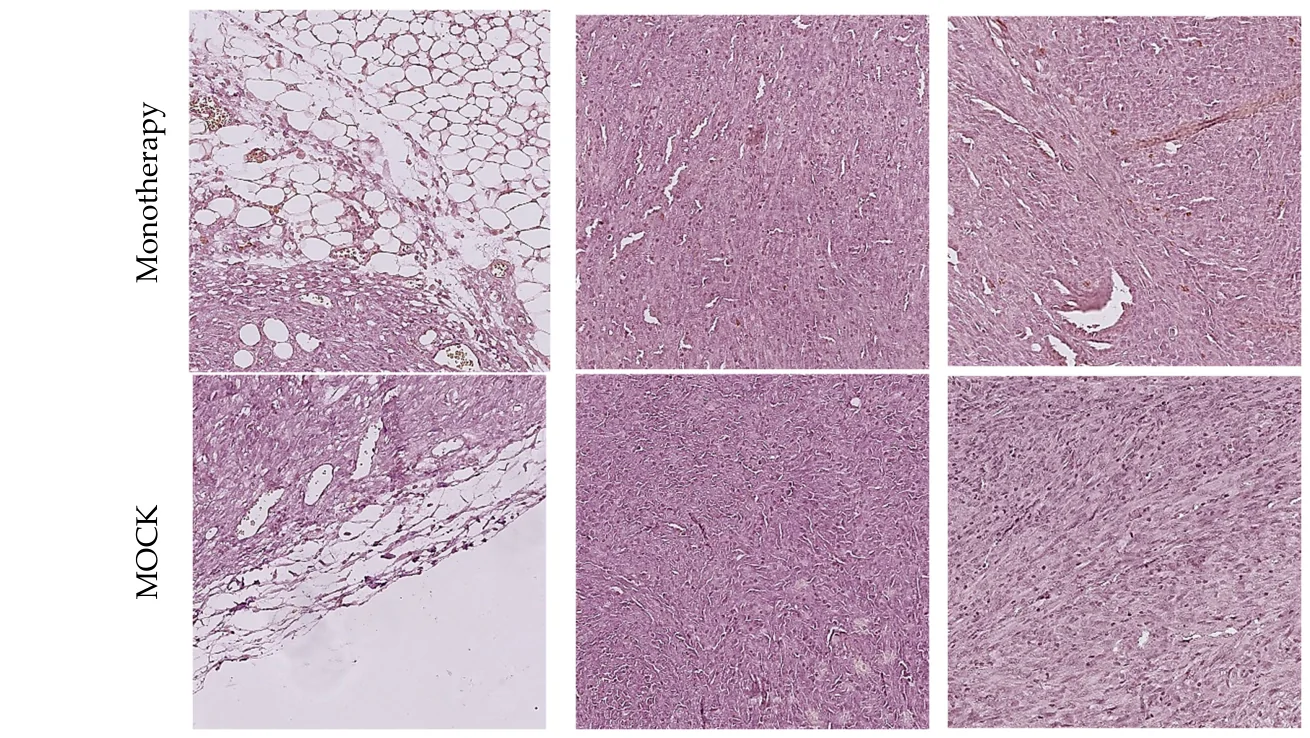
Representative IHC images showing CD3^+^, CD4^+^, and CD8^+^ cells in the peritumoral area, intratumoral area, and tumor stroma on day 14 in different groups. Immunohistochemical staining was performed using a standard automated protocol on a Ventana Benchmark XT staining platform (Ventana Medical Systems) with the ultraView Universal DAB Detection Kit (Ventana). The following primary antibodies were used: anti-CD3 (Abcam, cat. no. ab135372), anti-CD4 (Abcam, cat. no. ab237722), and anti-CD8 (Abcam, cat. no. ab217344). Immunostaining was performed for all treatment and control groups at each time point.

**Figure 11:**
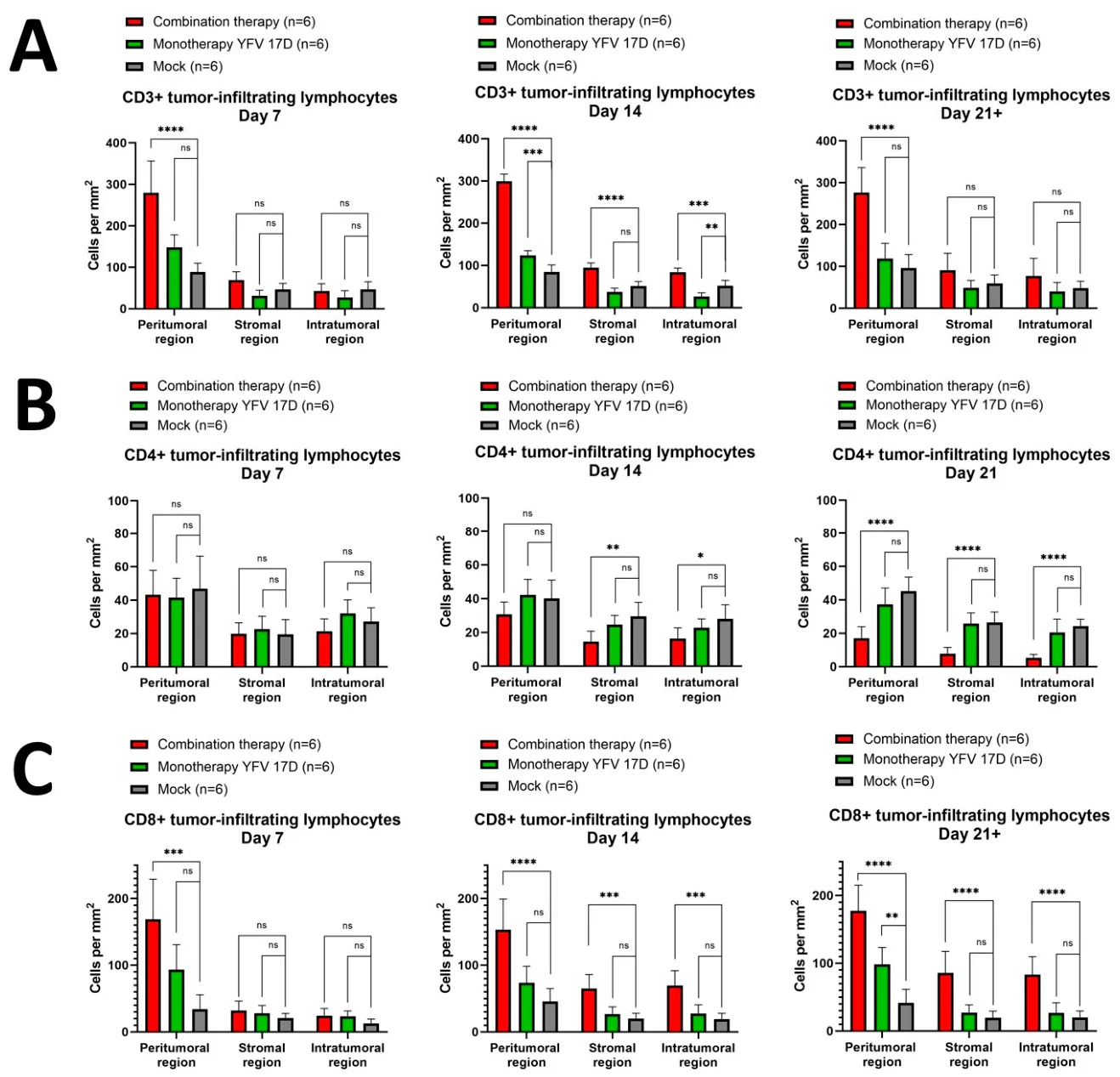
Quantitative analysis of CD3^+^ (**A**), CD4^+^ (**B**) and CD8^+^ (**C**) tumor-infiltrating lymphocytes in peritumoral, stromal and intratumoral regions on days 7,14 and 21 in control (mock), 17D monotherapy and combination therapy groups (cells per mm^2^, mean ± SEM, n = 6, * *p* < 0.05; ** *p* < 0.01; *** *p* < 0.001; **** *p* < 0.0001; ns—not significant). Quantitative analysis of immunohistochemical staining was carried out using QuPath software. Positive cells were quantified as the number of immunoreactive cells per 1 mm^2^ of tissue area. For each marker, cell density was assessed in representative tumor sections using the same analysis settings across all groups to ensure comparability.

CD3 is a marker reflecting the overall degree of T-lymphocyte infiltration and the involvement of adaptive immunity in the tumor microenvironment. All tumor samples from the study groups contained CD3-positive cells, primarily of immune origin, located in the peritumoral zone and, to a lesser extent, in the stroma and parenchyma (Figure 10 and Figure 11A). The number of CD3-positive cells was directly dependent on the treatment, as well as on the time points over the treatment course. In tumors from animals in the control group (mock), CD3-positive cells were detected in small numbers across all time points and formed a low-density infiltration, located in the stroma and in the parenchyma. In this group, the number of CD3-positive cells remained virtually unchanged over time compared to other groups. In the 17D monotherapy group, on days 7, 14, and 21 after intratumoral administration of YFV 17D, the number of CD3-positive cells in tumors was 1.5-fold lower than in the control group (mock). Lymphocytes were mainly localized in the peritumoral zone, and also in equal numbers in the stroma and parenchyma. The most significant were the changes in the number of CD3-positive lymphocytes in the combination therapy group. On day 7, it drastically increased 2.5-fold compared to the control group (mock), with CD3-positive lymphocytes being localized in the peritumoral zone. Throughout the experiment, CD3-positive lymphocytes tended to increase. On day 21, their total number peaked and was 3-fold higher than in the control group (mock). Analysis of CD3-positive lymphocyte distribution indicated their prevalence in the samples from the combination therapy group throughout the experiment, localizing mainly in the peritomoral zone but also migrating into the stroma and parenchyma, which indicated early T-cell response activation.

CD4 is a marker of T-helper lymphocytes, which comprise the regulatory component in the immune infiltrate. In the studied samples, their number tended to decrease both between groups, especially in the combination therapy group, and across time points (Figure 10 and Figure 11B). The highest number of CD4^+^ cells was detected in tumors from animals in the control group (mock) across all time points. They formed high-density infiltrates, located in both the stroma and parenchyma. On day 7 after intratumoral administration of YFV 17D, the number of CD4-positive cells was approximately the same in all groups, regardless of their location. On day 14 in the 17D monotherapy group, the number of CD4-positive cells remained the same, and on day 21, no changes were observed either. The CD4-positive lymphocyte count was the lowest in the combination therapy group. On the 7th day, their number was slightly less compared to the control group (mock) in the parenchyma. Throughout the experiment, CD4-positive lymphocytes tended to decrease, and on day 21, their total numbers were minimal—4-fold lower than in the control group (mock). In several animals, CD4-positive lymphocytes were virtually absent, with few occasional CD4-positive cells located diffusely in the stroma. Analysis of CD4-positive lymphocyte distribution indicates their prevalence in samples from the control group (mock) throughout the experiment, which does not exclude a T-reg immune status. However, their virtual absence in the combination therapy group suggests overcoming the tumor’s immunosuppressive state caused by the presence of CD4-positive/T-reg cells.

CD8 is a marker of cytotoxic T lymphocytes that reflects the severity of the effector antitumor immune response. In samples from all the groups, we observed CD8-positive cells located in both the stroma and parenchyma (Figure 10 and Figure 11C). The number of CD8-positive cells directly depended on both the treatment and time points. In tumor samples from animals in the control group (mock), we observed low-density infiltration with CD8-positive cells, which were present in low numbers across all time points. They were located in the stroma and, to a lesser extent, in the parenchyma. Their number remained virtually unchanged over time, compared to other groups. On day 7 after intratumoral administration of YFV 17D in the monotherapy group, the number of CD8-positive cells increased 2-fold. The lymphocytes were distributed in the stroma diffusely, closely to the tumor cells, filling the peritumoral zone. On days 14 and 21, the infiltrate density remained unchanged. In the combination therapy group, we observed high-intensity infiltration with CTL/CD8-positive cells. As early as day 14, the number of CD8-positive cells increased rapidly and was 3-fold higher than in the control group (mock). The lymphocytes infiltrated the stroma, peritumoral zone, and parenchyma. Throughout the experiment, CD8-positive lymphocytes tended to increase. On day 21, their count peaked and was 4-fold higher than in the control group. Immunophenotypic analysis revealed that throughout the experiment, CD8-positive lymphocytes, localized mainly in the stroma but then migrating into the tumor parenchyma, prevailed in samples from the combination therapy group. It indicates the gradual development of a cytotoxic antitumor response in the mice treated with the combination therapy.

Overall, immunohistochemical analysis of T-lymphocytes showed that in untreated PAN02 tumors, CD4-positive lymphocytes prevail, while CD8-positive cytotoxic lymphocytes, in contrast, are deficient, which corresponds to an immunologically unfavorable “cold” microenvironment of these tumors. Intratumoral administration of YFV 17D, followed by an increase in the CD8-positive cytotoxic lymphocytes count, caused partial immunomodulation of the tumor microenvironment, making it more “hot”. The combination therapy with YFV 17D and ruxolitinib caused an even stronger influx of cytotoxic lymphocytes, infiltrating the tumor parenchyma, and a significant decrease in CD4-positive (presumably immunosuppressive Treg) cells. These events led to the death of most cancer cells, which manifests as tumor tissue regression and a disruption of the tumor histoarchitectonics.

## 4. Discussion

PDAC remains one of the most lethal cancers, with a five-year survival rate of less than 12%, and is significantly resistant to both traditional chemotherapy and modern immunotherapeutic strategies. A key reason for the ineffectiveness of immunotherapy in PDAC is the immunologically “cold” status of the tumor microenvironment: a low number of cytotoxic CD8^+^ lymphocytes; prevalence of immunosuppressive regulatory cells, including CD4^+^/Treg cells and tumor-associated macrophages of the M2 phenotype, and dense desmoplasia, which impedes immune cell migration into the tumor. Oncolytic viruses are being studied as a potential means of remodeling the tumor microenvironment from “cold” to “hot,” thus converting PDAC into a state sensitive to immunotherapy. In this study, we investigated the combination of the attenuated 17D vaccine strain of yellow fever virus with the JAK1/JAK2 kinase inhibitor, ruxolitinib. We showed that this combination has significant potential for remodeling the tumor microenvironment. Remarkably, its mechanism of antitumor activity in vivo turned out to be fundamentally different from that observed in vitro.

### 4.1. In Vitro Response Heterogeneity: Basal Interferon Signaling Activity Is a Key Effectiveness Predictor for OV Therapy

Analysis of six PDAC cell lines with different mutation profiles revealed three main patterns of response to the combination therapy: synergistic enhancement of both viral replication rate and the virus-caused CPE (PAN02, PANC-1); lack of synergism and no additional benefit from the addition of ruxolitinib (MiaPaCa-2); and enhanced replication with the virus-caused CPE, with simultaneous CPE suppression (Capan-2, AsPC-1, BxPC-3). Despite all studied lines having different mutations in the *KRAS* and *TP53* genes (Table 1), we found that the cells’ response patterns were independent of these mutations’ presence, absence, or type. In contrast, the key predictors were the baseline level of JAK/STAT signaling activity and the functional integrity of the interferon barrier in the tumor line, which is consistent with published data regarding the key role of ISG-dependent antiviral response in PDAC resistance to oncolytic viruses [37].

In the case of MiaPaCa-2, with its extremely low basal levels of STAT1, STAT2, and IRF-9, the potency of the virus-caused lysis was unaffected by ruxolitinib, since this line virtually lacks an interferon barrier. This result served as an important negative control: the enhancing effect of ruxolitinib occurs exclusively in cells with functionally active JAK/STAT-dependent antiviral defense. Remarkably, we observed a phenomenon of increased virus replication concurrent with simultaneous CPE suppression in Capan-2, AsPC-1, and BxPC-3 cells, despite the differences in their *KRAS* and *TP53* mutation profiles. It is explained by the fact that JAK/STAT signaling simultaneously regulates activity of both ISG-dependent antiviral responses and pro-apoptotic pathways (PUMA, NOXA, and BAX), which are blocked by ruxolitinib.

### 4.2. Discrepancy Between In Vitro and In Vivo Results: The “Immunosuppressive Window” Mechanism

The most unexpected and significant observation of this study was the discrepancy between the effects of the combination therapy in vitro and in vivo. In PAN02 cells, the addition of ruxolitinib resulted in an increase in viral titer by approximately two orders of magnitude (10^2^ PFU/mL) and almost complete cell death by day 6. However, quantitative PCR analysis of tumor homogenates collected from PAN02 tumor-bearing syngeneic mice on days 3 and 5 after the start of therapy revealed no significant differences in YFV 17D RNA levels between the monotherapy and combination therapy groups. This discrepancy reflects the fundamental difference between a two-dimensional cell culture, lacking an immune microenvironment, and an immunocompetent in vivo model.

We hypothesize that the mechanism of this phenomenon is that a short-term blockade of JAK1/JAK2 kinases by ruxolitinib (24 and 3 h before and after YFV 17D administration) creates an “immunosuppressive window,” allowing the virus to readily infect and initiate replication in tumor cells before a full-fledged innate immune response takes effect. When the effect of ruxolitinib subsides and JAK/STAT signaling pathways are active again, the antiviral response takes effect, suppressing the virus’ replication. It is consistent with cytokine analysis data: the IFN-α level in tumor homogenates in the combination therapy group on day 3 was lower (~0.35 pg/mg) than in the monotherapy group (~0.97 pg/mg), which corresponds to a short-term but effective suppression of the early interferon response. A similar concept of a “temporary immunosuppressive window” has been described for other combinations of JAK inhibitors with oncolytic viruses. In particular, it was shown that short-term preadministration of ruxolitinib before intratumoral injection of oHSV increased the effectiveness of the initial stage of infection and subsequent activation of the CD8^+^ cell response [38]. In that case, long-term viral replication was also considered the secondary mechanism of the therapeutic effect.

### 4.3. The Contribution of the Adaptive Immune Response to the Antitumor Effect

One of the key findings of this study is that the antitumor efficacy of the combination of YFV 17D with ruxolitinib in the immunocompetent PAN02 model may be realized primarily through activation of the adaptive immune response rather than direct virus-caused oncolysis. The immunological data obtained in the study supported this finding. On day 10, the combination therapy group showed a pronounced peak in intratumoral IL-12 concentration (compared to ~1.0 pg/mg in the monotherapy group and ~0.85 pg/mg in the control group). IL-12 is a cytokine that plays a key role in Th1 polarization and priming of cytotoxic CD8^+^ lymphocytes [39]. The increase in IL-12 observed in this study is consistent with activation of a pro-inflammatory immune response. We suggest that the increase in IL-12 leads to dendritic cell activation, macrophage polarization, and CD8^+^ T-cell priming. However, these were not directly assessed in our study, and therefore, IL-12 should be interpreted here as an indirect indicator of immune activation, while the downstream functional consequences of this cytokine remain to be determined in future studies.

Immunohistochemistry data confirmed this hypothesis. By day 21, the number of CD8^+^ lymphocytes in tumors in the combination group was 4-fold higher than in the control and monotherapy groups, while the number of CD4^+^ cells, including presumably Tregs, decreased by approximately 4-fold. This dynamic indicates the overcoming of the tumor’s immunosuppressive “cold” state. Previously, a similar pattern (a simultaneous increase in CD8^+^ CTL and a decrease in CD4^+^/Treg) was described for a combination of ruxolitinib with oHSV, where CD8^+^ cells were found to be necessary and sufficient for the antitumor effect in vivo [38]. Importantly, in our study, increased infiltration with CD3^+^/CD8^+^ cells was consistent and continued up to day 21 of observation, which corresponds to the formation of a stable clonal expansion of cytotoxic lymphocytes rather than a short-term stress response.

The presence of neutralizing antibodies to YFV 17D in protective titers in all animals from both groups without statistically significant differences between them confirms that a short-term JAK/STAT blockade does not suppress the subsequent formation of a specific immune response. Undetectable cytokine levels in the serum samples and the absence of YFV 17D RNA in the brain and spleen of all animals, proven by ELISA and PCR analyses, further confirm the absence of neuroinvasion and systemic spread of the virus and the safety of the combination therapy.

### 4.4. Limitations and Future Research

It should be noted that PAN02, due to the species-specific nature of the NS5-STAT2 interaction (the virus does not inhibit mouse STAT2 but effectively inhibits human STAT2), does not fully reflect the molecular mechanisms occurring in human cells, where YFV 17D overcomes the interferon barrier more effectively. JAK/STAT pathway activity was not directly measured in vitro; instead, we relied on published data for the relevant cell lines. In vivo, we likewise did not directly assess JAK/STAT signaling, and our assumption of pathway inhibition is based on IFNa measurements. We also acknowledge that the panel of cytokines studied was limited—in particular, important mediators such as IFN-γ and Granzyme B were not evaluated. Thus, further research is needed to investigate the antitumor effect of the proposed therapy in orthotopic PDAC models, as well as its combination with immune checkpoint inhibitors. Furthermore, a detailed assessment of distinct lymphocyte phenotypes represents a promising direction for future research. In particular, analysis of the distribution of T-helper and T-regulatory subpopulations among CD3-positive immune cells, as well as CD8-positive cytotoxic lymphocytes, may prove especially informative in delineating the anti-tumor immune response across various models of PDAC. Moreover, synergism with immune checkpoint inhibitors has previously been demonstrated for YFV 17D, and taken together, this strategy could further enhance the CTL-dependent antitumor response demonstrated in this study.

## 5. Conclusions

This study demonstrates that the combination of the oncolytic vaccine strain YFV 17D with the JAK1/JAK2 inhibitor ruxolitinib has significant antitumor potential in PDAC. We suggest that one of the mechanisms may be activation of the adaptive immune response. Direct oncolysis may also contribute to it, but we believe, to a lesser extent. Analysis of six PDAC cell lines with different mutation profiles revealed three main patterns of response to the combination therapy: synergistic enhancement of both viral replication rate and the virus-caused CPE (PAN02, PANC-1); lack of synergism and no additional benefit from the addition of ruxolitinib (MiaPaCa-2); and enhanced replication with the virus-caused CPE suppression (Capan-2, AsPC-1, and BxPC-3). The key predictor of the response type was the baseline level of JAK/STAT signaling activity and the functional integrity of the cellular interferon barrier rather than mutation profiles of the cells studied. In the immunocompetent syngeneic PAN02 model, the combination therapy increased median survival by more than 2-fold compared with the control group (*p* < 0.0001), with partial or complete tumor lysis achieved in 5 out of 8 animals (62.5%). PCR analysis of tumor homogenates revealed no differences in viral RNA levels between the monotherapy and combination groups, ruling out enhanced in vivo replication as the primary mechanism of the antitumor effect. Instead, a short-term JAK/STAT blockade created an “immunosuppressive window,” enhancing the effectiveness of the initial phase of viral infection. Consequently, it led to immunogenic tumor cell death followed by an increase in intratumoral IL-12 levels, pronounced CTL infiltration (a 4-fold increase in CD8^+^ cells by day 21), and a decrease in CD4^+^ cells with a putative Treg phenotype. Together, it remodulated the tumor microenvironment state from immunologically “cold” to “hot.” Moreover, the combination therapy did not induce a systemic cytokine storm, neuroinvasion, or virus dissemination, which suggests a favorable safety profile. The titers of neutralizing antibodies to YFV 17D did not differ between the combination therapy and monotherapy groups, further confirming the safety of the proposed ruxolitinib administration regimen. The obtained data support the concept of personalized patient selection based on the assessment of basal JAK/STAT activity in the tumor and open up prospects for further optimization. This can be achieved in further research on orthotopic PDAC models and in combination with immune checkpoint inhibitors.

## Figures and Tables

**Table 1:** General characteristics of pancreatic cancer cell lines.

Cell line	Species Origin	*KRAS*		*TP53*		*SMAD4* Mutation	*CDKN2A* Mutation	Basal Activity of JAK/STAT	Viral Permissivity	IFNAR-1/2c on the Cell Surface	Ref.
		Mutation	Mutation Type	Mutation	Mutation Type						
PAN02	mouse	WT	WT	WT	WT	SMAD4-null status	WT	moderate	moderate	no data	[13,14]
PANC-1	human	G12D (hetero)	missence	R273H (hetero)	missence	WT	homo deletion	moderate	moderate	low	[15,16]
MiaPaCa-2	human	G12C (homo)	missence	R248W (homo)	missence	WT	homo deletion	very low	very high	moderate	[17,18,19]
Capan-2	human	G12V (homo)	missence	WT	WT	WT	homo deletion *	high	very low	high	[20,21,22,23]
AsPC-1	human	G12D (homo)	missence	C135AfsTer35 (p53-null)	frameshift	WT *	homo deletion	high	low	moderate	[16,24,25]
BxPC-3	human	WT	WT	Y220C (homo)	missence	homo deletion	homo deletion	high	low	high	[22,26,27]

* Data are contradictory.

**Table 2:** YFV 17D RNA levels in tumor, brain, and spleen homogenates in the combination therapy and monotherapy groups. * YFV 17D titers (PFU) in tumor homogenates after Vero cells were infected.

Group	YFV 17D RNA (copies/mg) and PFU(Vero) in the Tumors on Day 3 Post-Therapy			YFV 17D RNA (copies/mg) and PFU(Vero) in the Tumors on Day 5 Post-Therapy		
Combination therapy	10^2^	10^3^	10^2^	10^2^	10^2^	10^1^
	10 *	10^2^ *	10 *	10 *	10 *	1 *
YFV 17D monotherapy	10^3^	10^1^	10^2^	0	10^1^	10^2^
	10^1^ *	0 *	10^1^ *	0 *	0 *	10 *
	**YFV 17D RNA in the Brain After Euthanasia (n = 8)**			**YFV 17D RNA in the Spleen After Euthanasia (n = 8)**		
Combination therapy	Negative			Negative		
YFV 17D monotherapy	Negative			Negative		

## Data Availability

The original contributions presented in this study are included in the article. Further inquiries can be directed to the corresponding author.

## Abbreviations

The following abbreviations are used in this manuscript:

PDAC	Pancreatic Ductal Adenocarcinoma
YFV 17D	Yellow Fever Virus strain «17D»
JAK/STAT	Janus Kinase and Signal Transducer and Activator of Transcription
NS5	Viral nonstructural protein 5
hSTAT2	Human STAT2 protein
mSTAT2	Murine STAT2 protein
ISGs	Interferon-stimulated genes
MxA	Myxovirus resistance protein A
OAS	2′–5′ oligoadenylate synthetase gene
ATP	Adenosine Triphosphate
IFNAR 1/2c	Interferon Alpha And Beta Receptor Subunit 1/2c
TYK2	Tyrosine Kinase 2
ISGF3	Interferon-Stimulated Gene Factor 3
IRF9	Interferon Regulatory Factor 9
MOI	Multiplicity of infection
RTCA	Agilent’s Real-Time Cell Analysis
CPE	Cytopathic Effect
PUMA	p53 upregulated modulator of apoptosis
NOXA	Latin for “damage”, of the Bcl-2 protein family
BAX	BCL2-associated X protein
PAMPs	Pathogen-Associated Molecular Patterns
DAMPs	Damage-Associated Molecular Patterns
TAAs	Tumor-Associated antigens
